# Urban Green Forest Tree Diversity and Its Contribution to Timișoara’s Landscape Architecture

**DOI:** 10.3390/plants15040603

**Published:** 2026-02-13

**Authors:** Alina-Maria Țenche-Constantinescu, Cristian Berar, Emilian Onisan, Ioan Sărac, Sorina Popescu, Ciprian George Fora, Dorin Camen, Daniel Ond Turcu, Romuald Csaba Lorinț, Cristian-Iliuță Găină, Adina Horablaga, Cosmin Alin Popescu, Mihai Valentin Herbei, Lucian Dragomir, Virgil Dacian Lalescu

**Affiliations:** 1 Faculty of Engineering and Applied Technologies, University of Life Sciences “King Mihai I” from Timisoara, 119 Calea Aradului Street, 300645 Timisoara, Romania; alinaconstantinescu@usvt.ro (A.-M.Ț.-C.); cristianberar@usvt.ro (C.B.); ioansarac@usvt.ro (I.S.); sorinapopescu@usvt.ro (S.P.); ciprian.fora@usvt.ro (C.G.F.); dorincamen@usvt.ro (D.C.); 2 Faculty of Urban Planning and Architecture, Technical University of Moldova, 2004 Chisinau, Moldova; 3 Faculty of Agriculture, University of Life Sciences “King Mihai I” from Timisoara, 119 Calea Aradului Street, 300645 Timisoara, Romania; emilian.onisan@usvt.ro (E.O.); adinahorablaga@usvt.ro (A.H.); cosmin_popescu@usvt.ro (C.A.P.); mihaiherbei@usvt.ro (M.V.H.); luciandragomir@usvt.ro (L.D.); 4 Research-Development and Experimentation-Production Station (RDEPS) Timișoara, 8 Padurea Verde Street, 300310 Timisoara, Romania; daniel.turcu@icas.ro; 5 Mining Faculty, University of Petrosani, 332006 Petrosani, Romania; csabalorint@upet.ro; 6 Faculty of Management and Rural Tourism, University of Life Sciences “King Mihai I” from Timisoara, 119 Calea Aradului Street, 300645 Timisoara, Romania; cristian.gaina@gapa.ro; 7 Faculty of Food Engineering, University of Life Sciences “King Mihai I” from Timisoara, 119 Calea Aradului Street, 300645 Timisoara, Romania

**Keywords:** urban forest, biodiversity, landscape design and management

## Abstract

Urban forests serve as representations of nature within city landscapes. Green Forest, spanning 5,198,412 square meters, has been incorporated into the Municipality of Timișoara’s public domain and designated as a forest park. This fact increased green space per capita and enriched biodiversity within Timișoara’s landscape architecture. This study explores the diversity of Green Forest trees and highlights their contribution to the urban landscape. Statistical methods, including comparative and linear relationships analyses, were employed to assess significant variations in the dendrometric parameters of the analyzed tree species: mean tree height, mean trunk diameter at breast height (DBH), tree age, and stand density. Principal Component Analysis (PCA) and cluster analysis were applied to uncover underlying patterns in the data. Using ArchiCAD and Lumion, high-quality 3D visual representations were developed for an ecological education area, an active recreation region, and a passive recreation area within Green Forest. Due to their morphological characteristics and phenotypic traits, the predominant tree species include *Quercus robur*, *Quercus cerris*, *Quercus rubra*, *Fraxinus excelsior*, *Acer platanoides*, *Acer pseudoplatanus*, *Ulmus campestris*, and *Robinia pseudoacacia*, which contribute to Timișoara’s urban aesthetic. Moreover, the results of the dendrometric analysis provide a foundation for further research in urban ecology. A key practical application of this study is landscape design renderings, which provide detailed and realistic visualizations to effectively communicate the design and functionality of Green Forest’s spaces. If implemented, these developments will encourage public engagement with nature, promoting mental and physical well-being within the community.

## 1. Introduction

As the world faces the adverse impacts of climate change and elevated pollution, the need for effective action plans to mitigate these pressures has become urgent. Consequently, societal demand for reliable forest-related information has grown substantially. This demand prompted the establishment of the Collaborative Forest Resources Questionnaire (CFRQ), a global joint forest reporting mechanism, at the turn of the millennium [1,2]. According to the Global Forest Resources Assessment 2025 (FRA 2025) of the Food and Agriculture Organization of the United Nations (FAO), forests cover 4.14 billion hectares, approximately one-third of the planet’s land area, equivalent to about 0.5 hectares per person. There is a global, collaborative effort to support significant international commitments, including the 2030 Agenda for Sustainable Development, the Paris Agreement on Climate Change, the Kunming–Montreal Global Biodiversity Framework, and the United Nations Strategic Plan for Forests 2017–2030 [3]. Moreover, the European Forest Strategy aims to unlock forests’ potential for the future. European Union (EU) frameworks, such as the European Green Deal and the EU Biodiversity Strategy for 2030 (European Commission 2020), recognize the central and multifunctional role of forests and their contribution to achieving the EU’s greenhouse gas emission reduction target of at least 55% by 2030, as well as climate neutrality by 2050, as established under the European Climate Law. In this context, numerous studies worldwide focus on forest stands, examining their ecological [4,5,6,7], silvicultural [8,9,10], and socio-economic characteristics [11,12,13,14,15,16].

Romania’s forests, known for their rich biodiversity and ecological significance, face unprecedented challenges due to climate change. In their study, Stamin, F.D., and Cosmulescu, S. (2025) [17], proved that species such as beech (*Fagus sylvatica*), spruce (*Picea abies*), oak (*Quercus* spp.), and fir (*Abies alba*) are particularly vulnerable to these changes, while Chivulescu, S. et al. (2025), concluded that sessile oak (*Quercus petraea*) and hornbeam (*Carpinus betulus*) face greater challenges due to changing aridity patterns [18].

Rapid urbanization is impacting Romanian forests located near major urban centers [19,20]. At the local scale, several studies have focused on periurban forests in Romania, but few of them have analyzed the structural diversity of these ecosystems, their species composition, succession, and resilience [21,22,23]. In other studies, information on biodiversity and forest structure was used to model the recreational functions of forests [24,25]. A recent article presents information on the structure, diversity, and health status of one of the main periurban forests in Bucharest [26]. It emphasizes its importance in providing specific ecosystem services to the urban agglomeration.

Urban forests, as representations of nature within city landscapes, provide both economic and social benefits while simultaneously maintaining ecological health through climate regulation, improved air quality, and habitat provision for biodiversity. Recent reports and studies highlight urban trees as vital tools for mitigating heat, underscoring their importance in building resilient, livable cities [27,28]. At the same time, the presence of old trees in urban areas depends strongly on tree management, as their persistence requires long-term protection and care, which is constrained by the availability of financial resources.

In Romania, significant spatial transformations in land use, including changes in forest cover, exert high pressure on natural ecosystems [29,30]. A substantial discrepancy exists between civil society’s expectations for forest policies and the outcomes delivered by current forest management practices. This discrepancy is also reflected in the noteworthy evolution of Green Forest, whose first recorded forest management plan dates back to 1860. Initially owned by the town of Timișoara, it was later designated as a state forest, and today it once again falls under municipal ownership. Written historical documents indicate that, during Turkish rule, a hunting forest already existed in this area by 1711. In one of the oldest available cartographic sources, Griselini’s map from 1776, old-growth woods are depicted in the Green Forest area, extending beyond the present-day boundary of Dumbrăviţa village. Moreover, an early 19th-century map depicts a large hunting forest in the Green Forest region and indicates that the current site of the Forestry School Group was once a hunting lodge [31].

At present, in the context of a rapidly growing metropolitan area, it is pivotal to implement the policies outlined in the 2023 Action Plan for Timișoara, a Green City. The incorporation of Green Forest into Timișoara’s green infrastructure as a forest park reflects a strategic shift in urban planning—one that prioritizes sustainability, climate change adaptation, and the enhancement of urban quality of life, as emphasized in the 2023 General Urban Plan (PUG). Although the forest already existed and its ecological role remained unchanged, the transfer of 5,198,412 square meters of woodland into the Municipality of Timișoara’s public domain increases the officially recorded green space per capita. It enables improved management, protection, and integration of this area into the city’s ecological planning and landscape architecture strategies.

This research investigates the tree diversity of Green Forest and its contribution to the landscape architecture of Timișoara, with the forest stand serving as the study area.

The specific aims of the research are: (i) to explore the diversity of tree species in the Green Forest, highlighting their role in enhancing urban landscapes; and (ii) to promote the forest’s ecological and recreational functions through proposed interventions, including the development of an ecological education zone, a passive/transit recreation area, and an active recreation zone within the Green Forest.

The positive relationship between species richness and area is a fundamental principle of ecology, suggesting that larger habitats tend to support more species. Although this principle underpins the ecological significance of incorporating a large area such as Green Forest into the urban landscape, our ability to fully capture this effect is constrained by the scope of the present study. Because biodiversity was evaluated solely through tree species composition, excluding shrubs, herbaceous plants, fungi, and faunal communities, the observed increase in species richness likely represents only a fraction of the diversity expected under the species–area relationship. Consequently, the true magnitude of biodiversity enhancement associated with the expanded urban green area is presumably higher than our results indicate. A more comprehensive assessment that integrates multiple taxonomic groups and functional traits would be necessary to fully quantify the ecological value and conservation potential of Green Forest.

Our findings contribute to knowledge on how the interspecific diversity and intraspecific variability of the tree individuals forming the Green Forest enrich Timișoara’s biodiversity, while also highlighting their role in embellishing the urban landscape. Our proposal to maximize the ecological and recreational functions of the Green Forest will enhance the value of the ecosystem services it provides to Timișoara. If implemented, these developments will encourage public engagement with nature, promote mental and physical well-being within the community, and serve as a response tool for the 2023 Action Plan for Timișoara, a Green City. Our results provide insights and transferable knowledge to improve urban landscapes and biodiversity management, and may prove useful in similar contexts worldwide.

## 2. Results

### 2.1. Tree Species Identified in Different Plots of Green Forest

The Green Forest is primarily composed of 24 tree species populations, grouped into 16 genera, as follows. Quercus includes pedunculate oak (*Quercus robur* L.) Q. rob, red oak (*Quercus rubra*) Q. rub, Turkey oak (*Quercus cerris*) Q. cer, and Hungarian oak (*Quercus frainetto*) Q. frai. Fraxinus is represented by common ash (*Fraxinus excelsior*) F. exc. The genus Acer contains field maple (*Acer campestre*) A. cam, Norway maple (*Acer platanoides*) A. pla, and sycamore maple (*Acer pseudoplatanus*) A. pse. Ulmus includes European field elm (*Ulmus minor*) U. min and wych elm (*Ulmus glabra*) U. gla. Robinia is represented by black locust (*Robinia pseudoacacia*) R. pse. Juglans contains white walnut (*Juglans cinerea*) J. cin and black walnut (*Juglans nigra*) J. nig. Prunus includes wild cherry (*Prunus avium*) P. avi. Carpinus is represented by hornbeam (*Carpinus betulus*) C. bet. Gleditsia includes honey locust (*Gleditsia triacanthos*) G. tri. Pyrus contains European wild pear (*Pyrus pyraster*) Py. pyr. Tilia is represented by silver linden (*Tilia tomentosa*) T. tom. Castanea includes sweet chestnut (*Castanea sativa*) Ca. sat. Picea contains Norway spruce (*Picea abies*) Pi. abi. Taxus includes English yew (*Taxus baccata*) T. bac. Pinus contains black pine (*Pinus nigra*) P. nig and Scots pine (*Pinus sylvestris*) P. syl. Finally, Pseudotsuga is represented by Douglas fir (*Pseudotsuga menziesii*) Ps. Men, as shown in Table 1.

**Figure 1:**
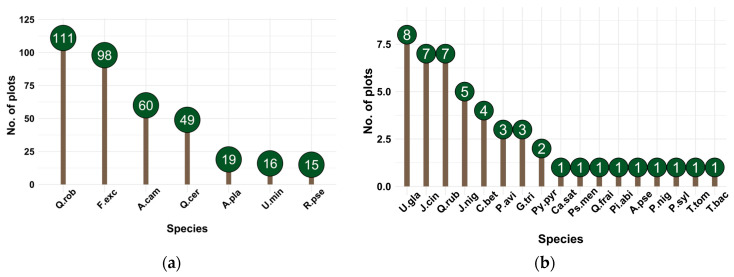
Spatial distribution of tree populations among Green Forest stand plots: (**a**) occurring in 111–15 plots; (**b**) occurring in 8–1 plots. Q. rob—*Quercus robur*, F. exc—*Fraxinus excelsior*, A. cam—*Acer campestre*, Q. cer—*Quercus cerris*, A. pla—*Acer platanoides*, U. min—*Ulmus minor*, R. pse—*Robinia pseudoacacia*, U. gla—*Ulmus glabra*, J. cin—*Juglans cinerea*, Q. rub—*Quercus rubra*, J. nig—*Juglans nigra*, C. bet—*Carpinus betulus*, P. avi—*Prunus avium*, G. tri—*Gleditsia triacanthos*, Py. pyr—*Pyrus pyraster*, Ca. sat—*Castanea sativa*, Ps. men—*Pseudotsuga menziesii*, Q. frai—*Quercus frainetto*, Pi. abi—*Picea abies*, A. pse—*Acer pseudoplatanus*, P. nig—*Pinus nigra*, P. syl—*Pinus sylvestris*, T. tom—*Tilia tomentosa*, T. bac—*Taxus baccata*.

Tree species populations occurring in the highest number of plots are associated with the following genera: Quercus, with Q. rob recorded in 111 plots, Q. cer in 49 plots, Q. rub in 7 plots, and Q. frai in one plot; Fraxinus with F. exc present in 98 plots; Acer with A. cam in 60 plots, A. pla in 19 plots, and A. pse in one plot; Ulmus, with U. min in 16 plots and U. gla in 8 plots; and Robinia with R. pse in 15 plots. Conversely, trees from the genera Taxus, Tilia, Picea, Castanea, and Pseudotsuga were identified in only one plot, as shown in Figure 1.

These species together create a unique ecosystem within the urban environment, enhancing it with a dynamic diversity of shapes and colors throughout the year.

### 2.2. Descriptive Statistics of Each Tree Species Identified—Green Forest Tree Diversity

Even in plots where trees are of the same species and age, they do not exhibit uniform development. This variation is influenced by genetic heredity and a heterogeneous environment, including microsite conditions that offer slightly better or worse living conditions within the same habitat. As a result, tree height, diameter, volume, and basal area vary among individuals. Consequently, each plot develops its own dimensional structure, a specific distribution of trees by height and diameter classes, and correspondingly, by volume or basal area [32]. This structural variability creates diversity within unity, contributing to the uniqueness of forest landscapes. A descriptive analysis was conducted on the variables mean age (A), mean trunk diameter at breast height (DBH), mean tree height (H), and mean consistency of stand densities (C) to assess central tendency, variability, and distributional shape. By assessing stand structure, this analysis offers insights into the physical characteristics of tree species populations within the Green Forest. The forest canopy is an important indicator used to measure stand density, with canopy cover typically associated with forest planning and inventories, while canopy closure is more commonly applied in ecologically oriented forest research [33]. The consistency or density of a forest stand strongly influences light-and-shadow dynamics by determining the amount of light penetrating the canopy, the spatial pattern of shadows, and the contrast and texture of light on the lower strata of the forest [34].

Higher density tends to reduce light transmission and increase shading, while lower density or gaps allow for more light to penetrate and create more varied light-and-shadow contrasts. These characteristics are purposefully used in landscape architecture to create diverse visual perspectives and to either conceal or highlight specific compositional focal points.

#### 2.2.1. Quercus Genus Tree Population Descriptive Statistics

Quercus genus tree population identified in Green Forest is represented by the following tree species: Pedunculate oak (*Quercus robur* L.)—Q. rob; Red oak (*Quercus rubra*)—Q. rub; Turkey oak (*Quercus cerris*)—Q. cer; Hungarian oak (*Quercus frainetto*)—Q. frai.

##### Pedunculate Oak (*Quercus robur* L.)—Q. rob

**Figure 2:**
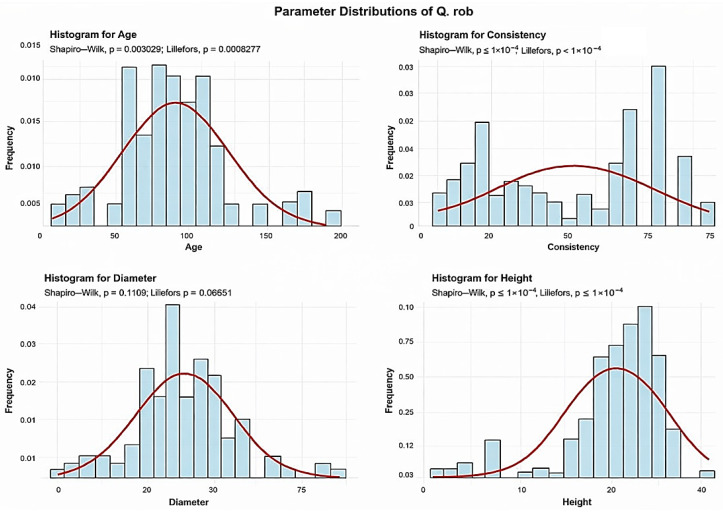
Pedunculate oak (*Quercus robur* L.)—Q. rob histograms of age, consistency, diameter, and height.

Descriptive analyses were conducted for the age, consistency, diameter, and height to summarize their central tendency, variability, and distributional characteristics within the sample, as shown in Figure 2.

The mean age of the Q. rob is 87 years. The standard deviation of the age of the Q. rob trees in the studied plots is 35.5 years. A total of 9 plots (8.11%) contain Q. rob with an average age of up to 30 years. Another 17 plots (15.32%) have Q. rob with an average age between 30 and 60 years. The largest group includes 40 plots (36.04%), where the average Q. rob age is between 60 and 90 years. In addition, 35 plots (31.53%) have Q. rob with an average age between 90 and 120 years. Beyond these dominant classes, we also observed 4 plots with average Q. rob ages ranging from 120 to 150 years, 5 plots with average Q. rob ages ranging from 150 to 180 years, and 1 plot with trees older than 180 years. Together, these oldest age classes represent less than 10% of the total.

The mean Q. rob consistency of stand-density values is 45.69, with a standard deviation of 25.33. Among all plots, 45 plots (40.54%) show degraded Q. rob consistency, 35 plots (31.53%) show discontinuous consistency, and 31 plots (27.93%) exhibit nearly full consistency.

Q. rob exhibits an average trunk diameter of 39.27 cm with a standard deviation of 16.10 cm. This means that while the average diameter is 39.27 cm, tree mean diameters can vary significantly, ranging from as small as 0.5 cm in plots where there is seedling regeneration, to as large as 86 cm in plots with mature, old-growth trees. In 34 plots (30.63% of the total), the average tree mean diameter of the Q. rob is up to 30 cm. In 70 plots (63.06%), the average diameter falls between 30 and 60 cm. Only 7 plots (6.31%) recorded average Q. rob diameters between 60 and 90 cm.

The average height of the Q. rob is 24.83 m, with a standard deviation of 6.51 m. This suggests that Q. rob trees are generally tall, though their heights show considerable variation—from the shortest individuals found in seedling-regeneration plots to the tallest, reaching 37 m in plots containing mature trees. In 100 plots (90.09% of the total), the average height is below 30 m, while only 11 plots (9.91%) recorded average Q. rob heights above 30 m.

##### Red Oak (*Quercus rubra*)—Q. rub

**Figure 3:**
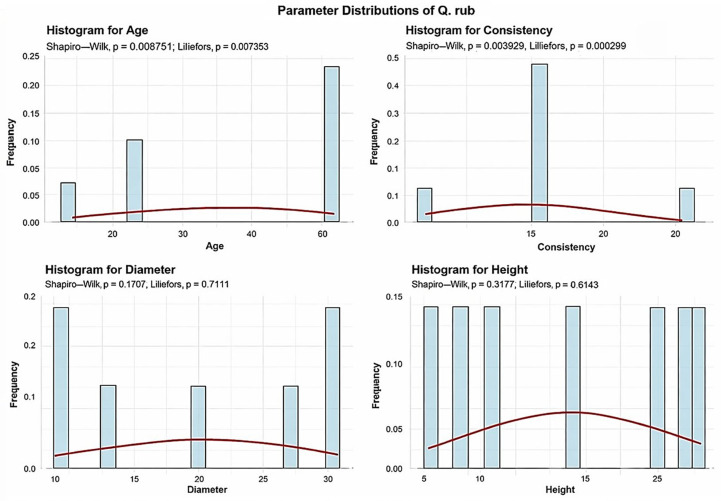
Red oak (*Quercus rubra*)—Q. rub histograms of age, consistency, diameter, and height.

Descriptive analyses were conducted for the age, consistency, diameter, and height to summarize their central tendency, variability, and distributional characteristics within the sample, as shown in Figure 3.

The mean age of the Q. rub is 43.57 years. The standard deviation of Q. rub age across the studied plots is 20.75 years, indicating moderate age-structure variability. Of the sampled plots, 3 plots (42.86%) contain Q. rub with an average age up to 30 years, while 4 plots (57.14%) have Q. rub with an average age between 30 and 60 years.

The mean Q. rub consistency of stand-density values is 10.42, with a standard deviation of 7.89. All seven plots exhibit degraded Q. rub consistency, indicating uniformly low values across the dataset.

The Q. rub exhibits an average trunk diameter of 20 cm, with a standard deviation of 8.87 cm. Across all 7 plots, the average tree mean diameter of the Q. rub falls within the range of 10 to 40 cm.

The average height of the Q. rub is 17 m, with a standard deviation of 7 m. Across the studied plots, the minimum average height recorded is 8 m, while the maximum reaches 25 m.

##### Turkey Oak (*Quercus cerris*)—Q. cer

**Figure 4:**
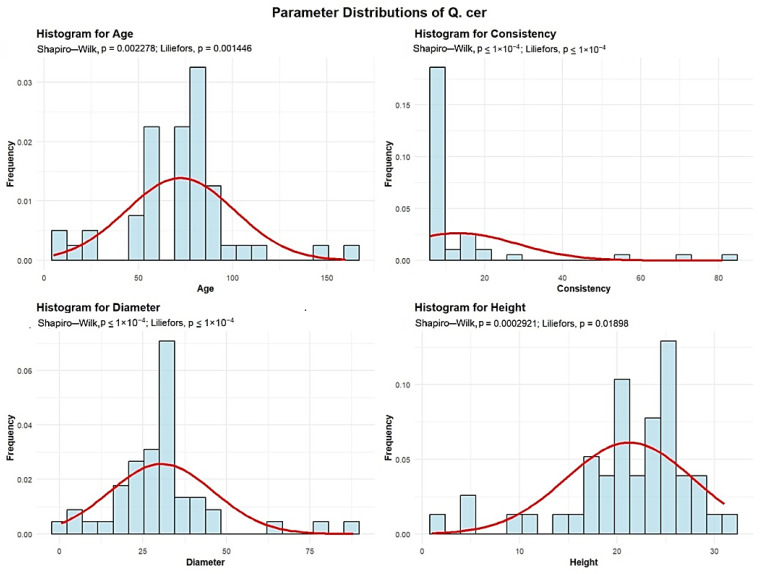
Turkey oak (*Quercus cerris*)—Q. cer histograms of age, consistency, diameter, and height.

Descriptive analyses were conducted for the age, consistency, diameter, and height to summarize their central tendency, variability, and distributional characteristics within the sample, as shown in Figure 4.

The mean age of the Q. cer is 72 years. The standard deviation of the age of the Q. rob trees in the studied plots is 28.74 years.

A total of 5 plots (10.2%) contain Q. cer with an average age of up to 30 years. Another 12 plots (24.49%) have Q. cer with an average age between 30 and 60 years. The largest group comprises 27 plots (55.1%), with average Q. cer age ranging from 60 to 90 years. Beyond these dominant classes, we identified 3 plots with average Q. cer ages between 90 and 120 years, 1 plot with ages between 120 and 150 years, and 1 plot with trees older than 150 years. Together, these oldest age categories represent 5% of the total.

The mean Q. cer consistency of stand-density values is 13.20, with a standard deviation of 15.18. Most plots, 46 plots (93.88%), exhibit degraded Q. cer consistency, while 1 plot (2.04%) shows discontinuous consistency, and 2 plots (4.08%) display nearly full consistency.

Q. cer exhibits an average trunk diameter of 30.60 cm with a standard deviation of 15.52 cm. This means that while the average diameter is 30.60 cm, tree mean diameters can vary significantly, ranging from as small as 0.5 cm in plots where there is seedling regeneration, to as large as 88 cm in plots with mature, old-growth trees. In 26 plots (53.06% of the total), the average tree mean diameter of the Q. cer is up to 30 cm. In 20 plots (40.82%), the average diameter ranges between 30 and 60 cm. Only 3 plots (6.12%) recorded average Q. cer diameters between 60 and 90 cm.

The average height of the Q. cer is 21.27 m, with a standard deviation of 6.51 m. This indicates that Q. cer trees are generally tall, though their heights vary considerably, from the shortest individuals in seedling regeneration plots to the tallest, which reach 31 m in plots with mature trees. In 48 plots (97.96% of the total), the average height is below 30 m, while only 1 plot (2.04%) recorded average Q. cer heights above 30 m.

##### Hungarian Oak (*Quercus frainetto*)—Q. frai

A population of *Quercus frainetto*—Q. frai was identified in only one plot within the Green Forest stand. Its characteristics are as follows: the mean age is 80 years, the mean diameter is 28 cm, the mean height is 20 m, and the mean consistency value is 7, indicating a degraded canopy.

#### 2.2.2. Fraxinus Genus Tree Population Descriptive Statistics

**Figure 5:**
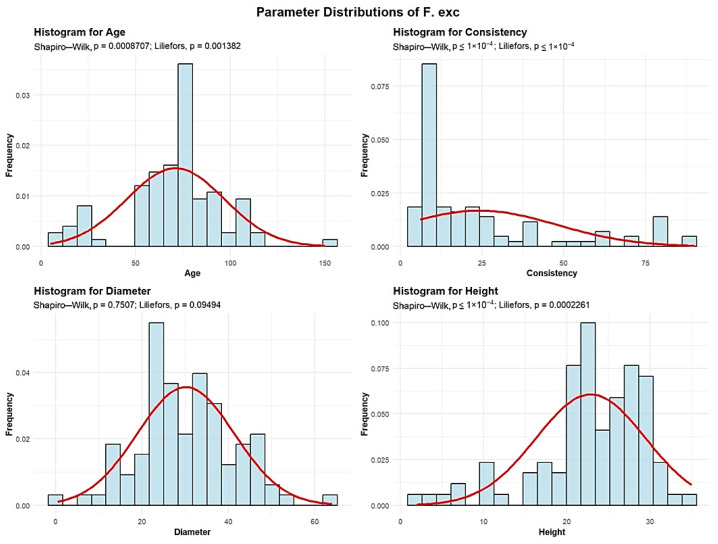
Common ash (*Fraxinus excelsior*)—F. exc histograms of age, consistency, diameter, and height.

The Fraxinus genus is represented within the Green Forest stand by the common ash (*Fraxinus excelsior*)—F. exc population. Descriptive analyses were conducted for the age, consistency, diameter, and height to summarize their central tendency, variability, and distributional characteristics within the sample, as shown in Figure 5.

The mean age of the F. exc is 71 years. The standard deviation of the age of the F. exc trees in the studied plots is 25.77 years.

A total of 12 plots (12.24%) contain F. exc with an average age of up to 30 years. Another 20 plots (20.41%) have F. exc with an average age between 30 and 60 years. The largest group includes 53 plots (50.08%), where the average F. exc age is between 60 and 90 years. In addition, 12 plots (12.24%) have F. exc with an average age between 90 and 120 years, and 1 plot containing trees older than 120 years.

The mean F. exc consistency of stand-density values is 24.10, with a standard deviation of 23.70. Most plots—76 plots (77.55%)—exhibit degraded F. exc consistency, while 12 plots (12.24%) show discontinuous consistency, and only 10 plots (10.20%) display nearly full consistency.

F. exc exhibit an average trunk diameter of 30.13 cm with a standard deviation of 11.21 cm. This means that while the average diameter is 30.13 cm, tree mean diameters can vary significantly, ranging from as small as 0.5 cm in plots where there is seedling regeneration, to as large as 64 cm in plots with mature trees. In 54 plots (55.1% of the total), the average tree mean diameter of the F. exc is up to 30 cm. In 43 plots (43.88%), the average diameter ranges from 30 to 60 cm. Only 1 plot recorded average F. exc diameters between 60 and 90 cm.

The average height of the F. exc is 22.88 m, with a standard deviation of 6.57 m. In most plots, 96 plots (97.96% of the total), the average height is below 30 m, while only 3 plots (2.04%) recorded average F. exc heights above 30 m.

#### 2.2.3. Acer Genus Tree Population Descriptive Statistics

The Acer genus tree population identified in Green Forest is represented by the following tree species: Field maple (*Acer campestre*)—A. cam, Norway maple (*Acer platanoides*)—A. pla, and sycamore maple (*Acer pseudoplatanus*)—A. pse.

##### Field Maple (*Acer campestre*)—A. cam

**Figure 6:**
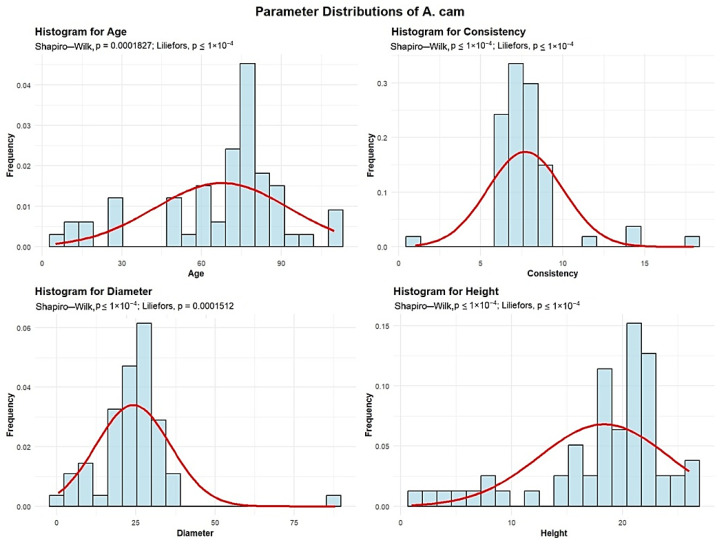
Field maple (*Acer campestre*)—A. cam histograms of age, consistency, diameter, and height.

Descriptive analyses were conducted for the age, consistency, diameter, and height to summarize their central tendency, variability, and distributional characteristics within the sample, as shown in Figure 6.

The mean age of the A. cam is 68 years. The standard deviation of A. cam age across the studied plots is 25.33 years, indicating considerable variability. A total of 9 plots (15%) contain A. cam with an average age up to 30 years, while 10 plots (16.67%) have A. cam with an average age between 30 and 60 years. The largest group comprises 36 plots (60%), with an average A. cam age of 60 to 90 years. Beyond these dominant classes, 5 plots (8.33%) were observed with average A. cam ages ranging from 90 to 120 years.

The mean A. cam consistency of stand-density values is 7.71, with a standard deviation of 2.29, meaning that all 60 plots exhibit degraded A. cam consistency.

A. cam exhibits an average trunk diameter of 24.14 cm with a standard deviation of 11.73 cm. This means that while the average diameter is 24.14 cm, tree mean diameters can vary significantly, ranging from as small as 0.5 cm in plots where there is seedling regeneration, to as large as 88 cm in plots with mature, old-growth trees.

In 51 plots (85% of the total), the average tree mean diameter of the A. cam is up to 30 cm. In 8 plots (13.33%), the average diameter ranged from 30 to 60 cm, while only 1 plot recorded average A. cam diameters between 60 and 90 cm.

The average height of the A. cam is 18.35 m, with a standard deviation of 5.85 m. This indicates that A. cam trees are generally tall, although their heights vary considerably, from the shortest individuals in seedling-regeneration plots to the tallest, reaching 26 m in plots with mature trees. In all 60 plots, the average A. cam height is below 30 m.

##### Norway Maple (*Acer platanoides*)—A. pla

**Figure 7:**
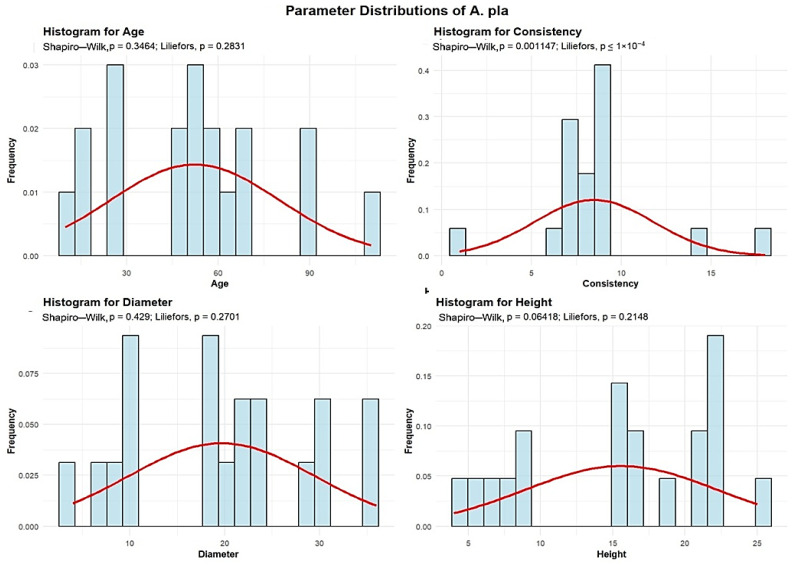
Norway maple (*Acer platanoides*)—A. pla histograms of age, consistency, diameter, and height.

Descriptive analyses were conducted for the age, consistency, diameter, and height to summarize their central tendency, variability, and distributional characteristics within the sample, as shown in Figure 7.

The mean age of the A. pla is 52.36 years. The standard deviation of the age of the Q. rob trees in the studied plots is 27.80 years.

A total of 6 plots (31.58%) contain A. pla with an average age of up to 30 years. The largest group includes 8 plots (42.11%) with A. pla averaging between 30 and 60 years. Additionally, 4 plots (21.05%) have A. pla with an average age between 60 and 90 years, and 1 plot contains trees older than 90 years.

The mean A. pla consistency of stand-density values is 8.47, with a standard deviation of 3.30, meaning that all 19 plots exhibit degraded A. pla consistency.

A. pla trees have an average trunk diameter of 19.68 cm, with a standard deviation of 9.78 cm, indicating substantial variation; diameters range from 4 cm to 36 cm in plots containing mature trees. In most plots, 17 plots (89.47% of the total), the average A. pla diameter is up to 30 cm, while only 2 plots (10.53%) have average diameters between 30 and 60 cm.

The average height of the A. pla is 15.57 m, with a standard deviation of 6.63 m. This indicates that A. pla trees are generally tall, although their heights vary considerably. In all 19 plots, the average A. pla height is below 30 m.

##### Sycamore Maple (*Acer pseudoplatanus*)—A. pse

A population of *Acer pseudoplatanus* (A. pse) was identified in only one plot within the Green Forest stand. Its characteristics are as follows: the mean age is 60 years, the mean diameter is 18 cm, the mean height is 17 m, and the mean consistency value is 9, indicating a degraded canopy.

#### 2.2.4. Ulmus Genus Tree Population Descriptive Statistics

Ulmus genus tree populations identified in the Green Forest are represented by the European field elm (*Ulmus campestris*)—U. min and the Wych elm (*Ulmus glabra*)—U. gla.

##### European Field Elm (*Ulmus campestris*)—U. min

**Figure 8:**
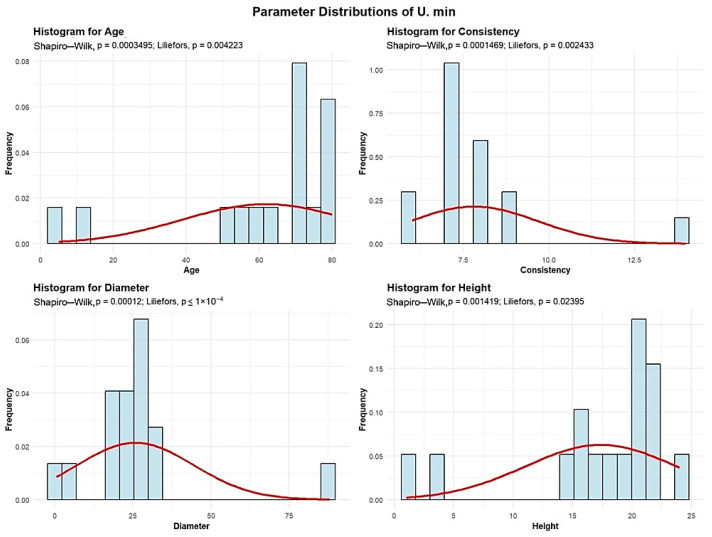
European field elm (*Ulmus campestris*)—U. min histograms of age, consistency, diameter, and height.

Descriptive analyses were conducted for the age, consistency, diameter, and height to summarize their central tendency, variability, and distributional characteristics within the sample, as shown in Figure 8.

The mean age of the U. min is 61.87 years. The standard deviation of the U. min tree ages in the studied plots is 35.5 years. A total of 2 plots (12.5%) contains U. min with an average age of up to 30 years. Another 3 plots (18.75%) have U. min with an average age between 30 and 60 years. The largest group includes 11 plots (68.75%), where the average U. min age is between 60 and 90 years.

The mean U. min consistency of stand-density values is 7.81, with a standard deviation of 1.86, meaning that all 16 plots exhibit degraded U. min consistency.

U. min exhibits an average trunk diameter of 25.78 cm, with a standard deviation of 18.68 cm. In most plots, 15 plots (93.75% of the total), the average U. min diameter is up to 30 cm, while only 1 plot has an average diameter between 60 and 90 cm.

The average height of the U. min is 17.37 m, with a standard deviation of 6.36 m, and the maximum recorded height is 24 m, meaning that in all 16 plots the average U. min height is below 30 m.

##### Wych Elm (*Ulmus glabra*)—U. gla

**Figure 9:**
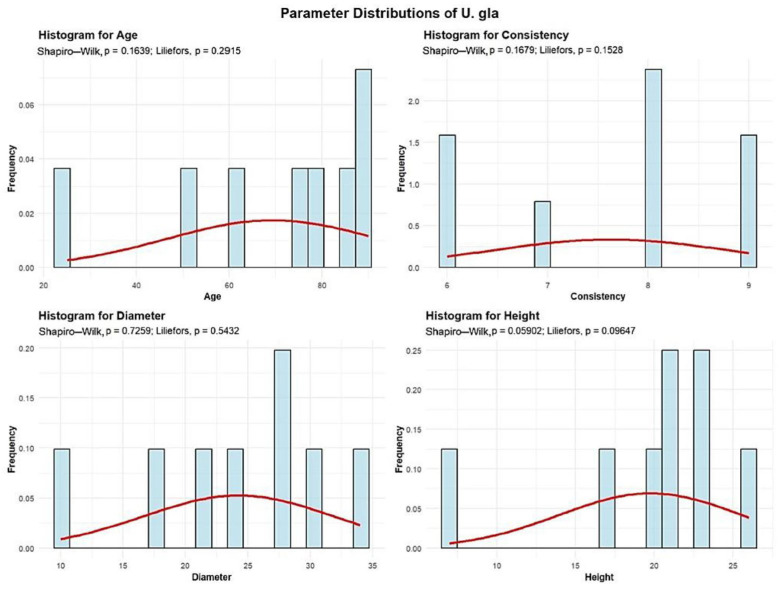
Wych elm (*Ulmus glabra*)—U. gla histograms of age, consistency, diameter, and height.

Descriptive analyses were conducted for the age, consistency, diameter, and height to summarize their central tendency, variability, and distributional characteristics within the sample, as shown in Figure 9.

The mean age of the U. gla is 69.37 years. The standard deviation of U. gla age across the studied plots is 22.9 years. A total of 2 plots (25%) contain U. gla with an average age between 30 and 60 years. The other 6 plots (75%) have U. gla with an average age between 60 and 90 years.

The mean U. gla consistency of stand-density values is 7.62, with a standard deviation of 1.18, meaning that all 8 plots exhibit degraded U. gla consistency.

U. gla exhibits an average trunk diameter of 24.25 cm, with a standard deviation of 5.7 cm.

In 7 plots, the average U. gla diameter is up to 30 cm, while only 1 plot shows an average diameter of 34 cm.

The average height of the U. gla is 19.75 m, with a standard deviation of 6.36 m, and the maximum recorded height is 26 m, meaning that in all 8 plots the average U. gla height is below 30 m.

#### 2.2.5. Robinia Genus Tree Population Descriptive Statistics

The Robinia genus is represented within the Green Forest stand by the black locust (*Robinia pseudoacacia*)—R. pse population.

**Figure 10:**
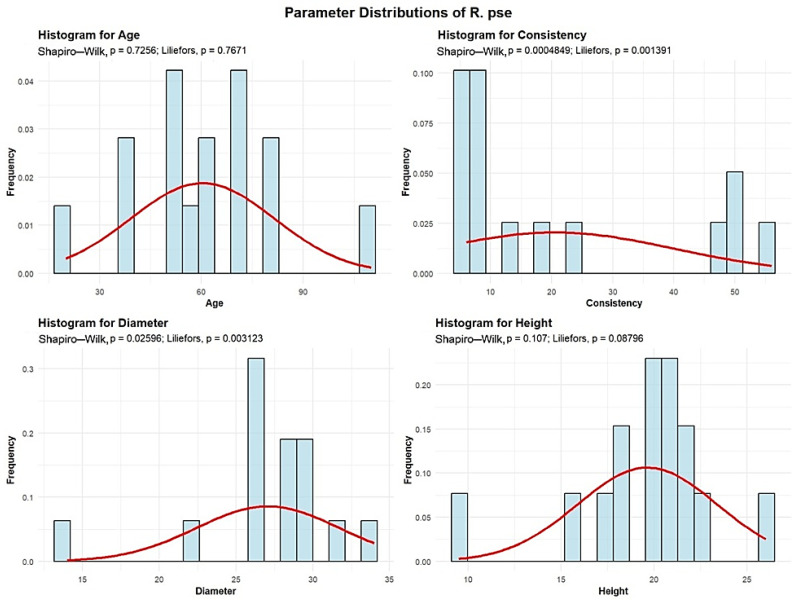
Black locust (*Robinia pseudoacacia*)—R. pse histograms of age, consistency, diameter, and height.

Descriptive analyses were conducted for the age, consistency, diameter, and height to summarize their central tendency, variability, and distributional characteristics within the sample, as shown in Figure 10.

The mean age of the R. pse is 60.33 years. The standard deviation of the ages of the R. pse trees is 21.25 years.

One plot (6.67%) contains R. pse with an average age of 20 years. The largest group includes 8 plots (53.33%) with R. pse averaging between 30 and 60 years. Another 5 plots (33.33%) have R. pse with an average age between 60 and 90 years, and one plot (6.67%) contains R. pse trees with an average age of 110 years.

The mean R. pse consistency of stand-density values is 20.53, with a standard deviation of 19.46. Among all plots, 11 plots (73.33%) show degraded R. pse consistency, while 4 plots (26.67%) exhibit nearly full consistency.

R. pse exhibit an average trunk diameter of 27.06 cm, with a standard deviation of 4.65 cm. The minimum recorded average trunk diameter is 14 cm, while the maximum reaches 34 cm. In most plots, 13 plots (86.67%), the average R. pse trunk diameter is up to 30 cm, whereas only 2 plots (13.33%) show average trunk diameters between 30 and 60 cm.

The average height of the R. pse is 19.63 m, with a standard deviation of 3.75 m. The minimum recorded average height is 9.5 m, while the maximum is 26 m, indicating that, across all 15 plots, the average R. pse height is below 30 m.

#### 2.2.6. Juglans Genus Tree Population Descriptive Statistics

Juglans genus tree populations identified in the Green Forest are represented by the White walnut (*Juglans cinerea*)—J. cin and the Black walnut (*Juglans nigra*)—J. nig.

##### White Walnut (*Juglans cinerea*)—J. cin

**Figure 11:**
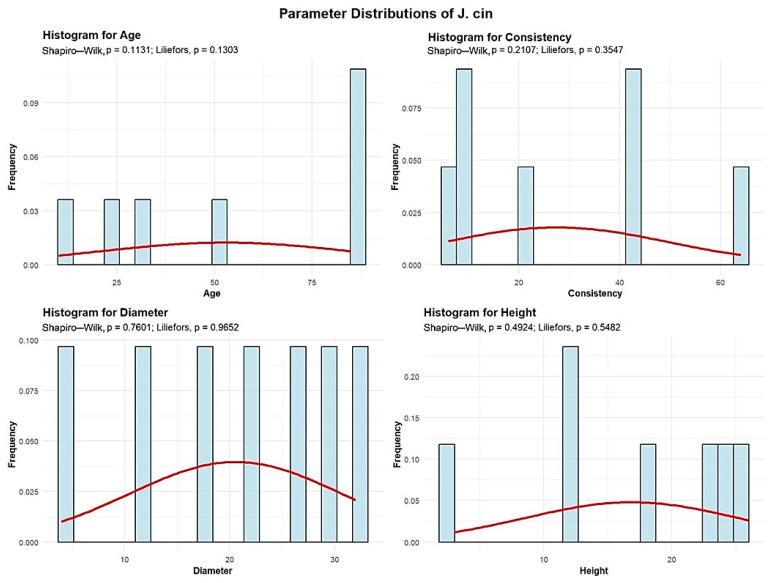
White walnut (*Juglans cinerea*)—J. cin histograms of age, consistency, diameter, and height.

Descriptive analyses were conducted for the age, consistency, diameter, and height to summarize their central tendency, variability, and distributional characteristics within the sample, as shown in Figure 11.

The mean age of the J. cin is 52.85 years. The standard deviation of J. cin age across the studied plots is 32.25 years. A total of 3 plots (42.86%) contain J. cin with an average age of up to 30 years. One plot (14.29%) contains J. cin with an average age of 50 years, while 3 plots (42.86%) have J. cin with an average age of 85 years.

The mean J. cin consistency of stand-density values is 27.42, with a standard deviation of 22.28. Among all plots, four plots (57.14%) show degraded J. cin consistency, while three plots (42.86%) show discontinuous consistency.

J. cin exhibits an average trunk diameter of 20.57 cm with a standard deviation of 10.04 cm. This means that tree mean diameters vary significantly, ranging from as small as 4 cm to as large as 32 cm.

The average height of the J. cin is 16.85 m, with a standard deviation of 8.29 m, and in all 7 plots, the average height remains below 30 m.

##### Black Walnut (*Juglans nigra*)—J. nig

**Figure 12:**
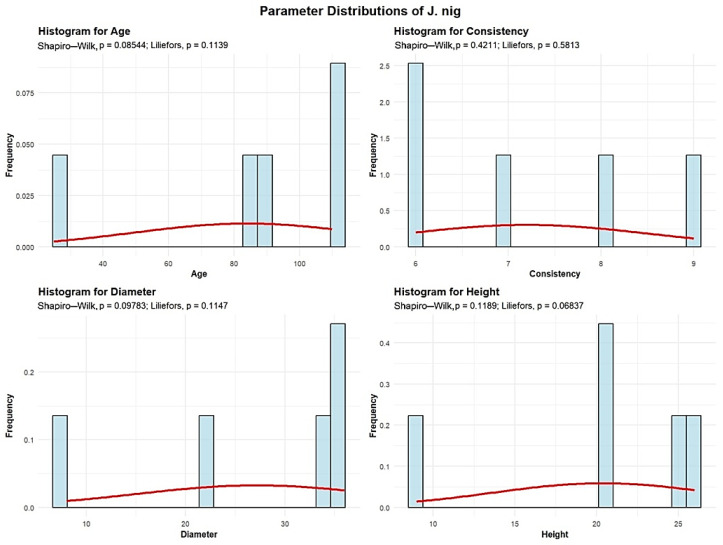
Black walnut (*Juglans nigra*)—J. nig histograms of age, consistency, diameter, and height.

Descriptive analyses were conducted for the age, consistency, diameter, and height to summarize their central tendency, variability, and distributional characteristics within the sample, as shown in Figure 12.

The mean age of the J. nig is 84 years. The standard deviation of J. nig age across the studied plots is 34.89 years. One plot contains J. nig with an average age of 25 years, while 4 plots (80%) contain J. nig with an average age greater than 80 years.

The mean J. nig consistency of stand-density values is 7.2, with a standard deviation of 1.30, meaning that all 5 plots show degraded J. nig consistency.

J. nig exhibits an average trunk diameter of 27.2 cm with a standard deviation of 12.21 cm. This means that tree mean diameters vary significantly, ranging from as small as 8 cm to as large as 36 cm.

The average height of the J. nig is 20.04 m, with a standard deviation of 6.76 m, and in all 5 plots, the average height remains below 30 m.

#### 2.2.7. Prunus Genus Tree Population Descriptive Statistics

The descriptive statistics for the Prunus population refer to Wild cherry (*Prunus avium*)—P. avi, which was identified in only three plots within the Green Forest stand.

The mean age of the P. avi is 73.33 years, with a standard deviation of 20.81 years. One plot contains P. avi with an average age of 50 years, while the other two plots contain P. avi with an average age greater than 80 years.

The mean P. avi consistency of stand-density values is 9.33, with a standard deviation of 4.16, meaning that all 3 plots show degraded P. avi consistency.

P. avi exhibits an average trunk diameter of 22 cm, with a standard deviation of 9.45 cm. The minimum average trunk diameter is 18 cm, while the maximum reaches 36 cm.

The average height of the P. avi is 19.33 m, with a standard deviation of 2.08 m. The minimum average height recorded is 17 m, and the maximum is 21 m.

#### 2.2.8. Carpinus Genus Tree Population Descriptive Statistics

The descriptive statistics for the Carpinus population refer to Hornbeam (*Carpinus betulus*)—P. avi, which was identified in only four plots within the Green Forest stand.

The mean age of the C. bet is 51.25 years, with a standard deviation of 32.24 years. In two plots, C. bet have an average age of up to 30 years, while in the other two plots, the average C. bet age ranges from 60 to 90 years.

The mean C. bet consistency of stand-density values is 6.75, with a standard deviation of 1.5, meaning that all 4 plots show degraded C. bet consistency.

C. bet exhibit an average trunk diameter of 23.5 cm, with a standard deviation of 10.63 cm. The minimum average trunk diameter is 12 cm, while the maximum reaches 36 cm. The average height of the C. bet is 16.25 m, with a standard deviation of 6.13 m. The minimum average height recorded is 10 m, and the maximum is 22 m.

#### 2.2.9. Gleditsia Genus Tree Population Descriptive Statistics

The descriptive statistics for the Gleditsia population refer to honey locust (*Gleditsia triacanthos)*—G. tri, which was identified in only three plots within the Green Forest stand. The mean age of the G. tri is 58.33 years, with a standard deviation of 2.88 years. The mean G. tri consistency of stand-density values is 7, with no variation across plots, meaning that all 3 plots show degraded G. tri consistency.

G. tri exhibits an average trunk diameter of 20.66 cm, with a standard deviation of 3.05 cm. The minimum average trunk diameter is 18 cm, while the maximum reaches 24 cm. The average height of the G. tri is 16.33 m, with a standard deviation of 1.52 m. The minimum average height recorded is 15 m, and the maximum is 18 m.

#### 2.2.10. Pyrus Genus Tree Population Descriptive Statistics

The descriptive statistics for the Pyrus population refer to European wild pear (*Pyrus pyraster*)—Py. pyr, which was identified in only two plots within the Green Forest stand. The mean age of the Py. pyr is 77.5 years.

The mean Py. pyr consistency of stand-density values is 8, with no variation across plots, indicating degraded Py. pyr consistency in the 2 plots.

Py. pyr exhibits an average trunk diameter of 26 cm; the minimum average trunk diameter is 22 cm, while the maximum reaches 30 cm.

The average height of the Py. pyr is 22.5 m, with a minimum of 19 m and a maximum of 26 m.

#### 2.2.11. Tilia Genus Tree Population Descriptive Statistics

The descriptive statistics for the Tilia population refer to silver linden (*Tilia argentea*)—T. tom, which was identified in only one plot within the Green Forest stand. Its characteristics are as follows: the mean age is 90 years, the mean trunk diameter is 36 cm, the mean height is 25 m, and the mean consistency value is 6, indicating a degraded canopy.

#### 2.2.12. Castanea Genus Tree Population Descriptive Statistics

The descriptive statistics for the Castanea population refer to sweet chestnut (*Castanea sativa*)—Ca. sat, which was identified in only one plot within the Green Forest stand. Its characteristics are as follows: the mean age is 90 years, the mean trunk diameter is 36 cm, the mean height is 21 m, and the mean consistency value is 6, indicating a degraded canopy.

#### 2.2.13. Picea Genus Tree Population Descriptive Statistics

The descriptive statistics for the Picea population refer to Norway spruce (*Picea abies*)—Pi. abi, which was identified in only one plot within the Green Forest stand. Its characteristics are as follows: the mean age is 90 years, the mean trunk diameter is 34 cm, the mean height is 24 m, and the mean consistency value is 6, indicating a degraded canopy.

#### 2.2.14. Taxus Genus Tree Population Descriptive Statistics

The descriptive statistics for the Taxus population refer to English yew (*Taxus baccata*)—T. bac, which was identified in only one plot within the Green Forest stand. Its characteristics are as follows: the mean age is 90 years, the mean trunk diameter is 34 cm, the mean height is 24 m, and the mean consistency value is 6, indicating a degraded canopy.

#### 2.2.15. Pinus Genus Tree Population Descriptive Statistics

Pinus genus tree populations identified in the Green Forest are represented by the Black pine (*Pinus nigra*)—P. nig and the Scots pine (*Pinus sylvestris*)—P. syl.

##### Black Pine (*Pinus nigra*)

P. nig was identified in only one plot within the Green Forest stand. Its characteristics are as follows: the mean age is 80 years, the mean trunk diameter is 32 cm, the mean height is 21 m, and the mean consistency value is 6, indicating a degraded canopy.

##### Scots Pine (*Pinus sylvestris*)

P. syl was identified in only one plot within the Green Forest stand. Its characteristics are as follows: the mean age is 90 years, the mean trunk diameter is 34 cm, the mean height is 24 m, and the mean consistency value is 6, indicating a degraded canopy.

#### 2.2.16. Pseudotsuga Genus Tree Population Descriptive Statistics

The descriptive statistics for the Pseudotsuga population refer to Douglas fir (*Pseudotsuga menziesii*)—Ps. men, which was identified in only one plot within the Green Forest stand. Its characteristics are as follows: the mean age is 90 years, the mean trunk diameter is 34 cm, the mean height is 24 m, and the mean consistency value is 6, indicating a degraded canopy.

### 2.3. Principal Component Analysis and Cluster Analysis

**Figure 13:**
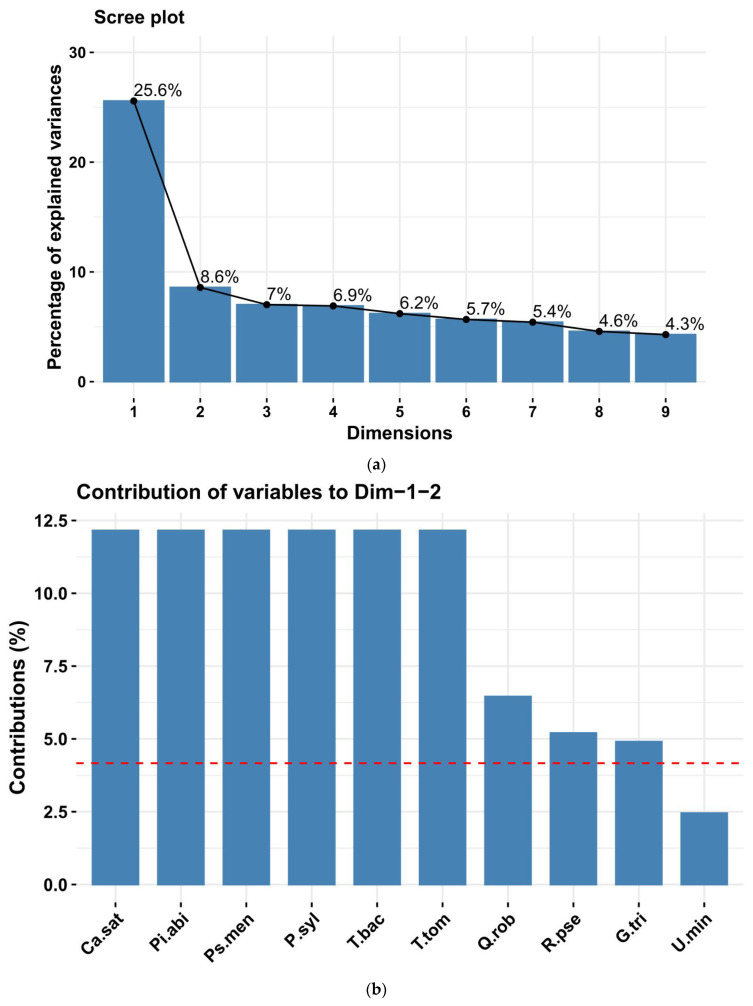

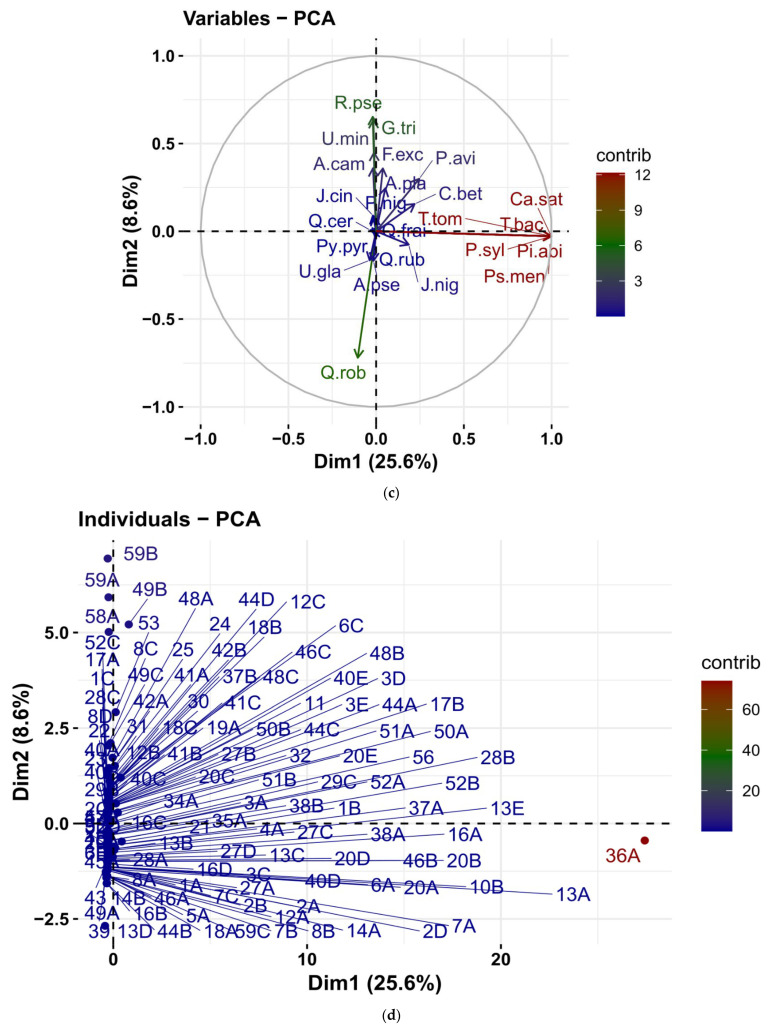
(**a**) Scree plot of PCA; (**b**) contribution of variables to the first and second dimensions of PCA, red dashed line indicates that the variables have the most significant contribution to the first two principal components of the PCA; (**c**) projection of variables on the plane spanned by the first and second principal components of PCA; (**d**) projection of individuals on the plane spanned by the first and second principal components of PCA. Red dashed line indicates that the variables have the most significant contribution to the first two principal components of the PCA.

The inertia of the first principal components indicates strong relationships among variables and suggests the number of components to examine. The first two principal components of analysis account for 34.2% of the total dataset inertia; this means that 34.2% of the individual’s total cloud variability is explained by the plane spanned by the first two principal components (Figure 13a). The first plane represents data variability well.

The first principal component is significant, accounting for 25.6% of the data variability (Figure 13a). This observation suggests that this axis carries great information. The most significant contributions to this component come from the following variables: Ca. sat, T. tom, Pi. abi, Ps. men, P. syl, and T. bac, which are highly correlated with this dimension. These variables could therefore summarize dimension 1 (Figure 13b,c). Dimension 1 opposes individuals such as 6C, 12B, 12C, 18B, 42B, 44D, and 16C (to the right of the graph, characterized by a strongly positive coordinate on the axis) to individuals characterized by a strongly negative coordinate on the axis (to the left of the graph). The group in which the individual 16C stands (characterized by a positive coordinate on the axis) shares high values for the variables U. gla, F. exc, and Q. cer (the variables are sorted from the strongest). The group, comprising individuals 6C, 12B, 12C, 18B, 42B, and 44D (characterized by a positive coordinate on the axis), exhibits high values for the variable F. exc and low values for the variable Q. rob (Figure 13d).

The second principal component accounts for 8.6% of the data variability (Figure 13a). The most important contribution of this component comes from the variables F. exc, U. min, R. pse, Q. rob, G. tri, P. avi, and A. cam, which are all highly correlated with this dimension (Figure 13b,c). These variables could therefore be used to summarize dimension 2.

**Figure 14:**
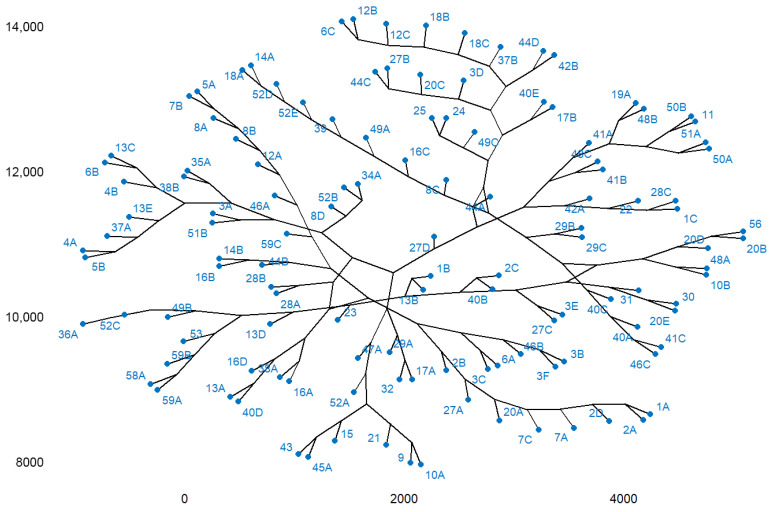
Dendrogram of the plots with respect to tree consistency according to their age, height, and diameter.

Cluster analysis is presented in Figure 14. This highlights the clustering of plots according to the studied variables in seven clusters as follows:

Cluster 1 includes the following plots: 1A, 1B, 1C, 2A, 2B, 2C, 2D, 3A, 3B, 3C, 3D, 3E, 3F, 4A, 4B, 5A, 5B, 6A, 6B, 6C, 7A, 7B, 7C, 8A, 8B, 8C, 8D, 9, 10A, 10B, 11, 12A, 12B, 12C, 13A, 13B, 13C, 13E, 14A, 14B, 15, 16A, 16B, 16C, 16D, 17A, 17B, 18A, 18B, 18C, 19A, 20A, 20B, 20C, 20D, 20E, 21, 22, 24, 25, 27A, 27B, 27C, 27D, 28A, 28B, 28C, 29A, 29B, 29C, 30, 31, 32, 34A, 35A, 37A, 37B, 38A, 38B, 39, 40A, 40B, 40C, 40D, 40E, 41A, 41B, 41C, 42A, 42B, 43, 44A, 44B, 44C, 44D, 45A, 46A, 46B, 46C, 47A, 48A, 48B, 48C, 49A, 49C, 50A, 50B, 51A, 51B, 52A, 52B, 52D, 52E, 56, and 59C.

Cluster 2 includes plot 13D.

Cluster 3 includes plot 23.

Cluster 4 includes plot 36A.

Cluster 5 includes plot 49B.

Cluster 6 includes plot 52C.

Cluster 7 includes the following plots: 53, 58A, 59A, and 59B.

### 2.4. Landscape Architecture Design Proposals

**Figure 15:**
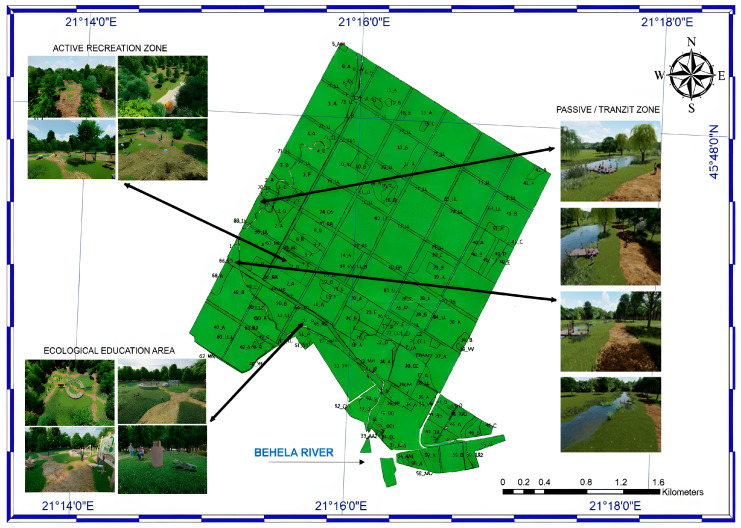
A three-zone landscape architecture framework is proposed for the Green Forest.

To maximize psychological and physical benefits for residents and to facilitate diverse human–nature interactions, three functionally distinct zones were delineated within Green Forest (Figure 15).

The proposed three-zone framework, ecological education, passive/transit use, and active recreation, follows established principles of urban forest planning and Nature-Based Solutions, which advocate the functional differentiation of green spaces based on intensity of use and ecological sensitivity. According to Davies et al. (2024), this zoning approach enables the coexistence of conservation objectives with recreational and educational functions by spatially concentrating higher-impact activities while safeguarding ecologically sensitive areas [35]. Similarly, Li et al. (2025) demonstrate that multifunctional zoning is widely applied in urban and periurban forest management to reduce ecological disturbance, enhance visitor experience, and ensure long-term ecological integrity alongside sustained social benefits [36].

Each zone is designed to support a specific mode of engagement with the forest landscape, thereby accommodating multiple user needs and experiential preferences.

The Ecological education area prioritizes scientific inquiry and knowledge dissemination, the Passive/Transit zone provides opportunities for contemplative experience, and the Active recreation zone supports more intensive forms of environmental engagement and physical activity. All zones were designed following natural vegetation lines, and we proposed using natural materials such as durable wood for urban furniture elements, the open library, signage, and playground equipment, and porous materials like tree bark and sand for pedestrian and bicycle paths, allowing water to infiltrate into the soil. Additionally, ecological toilets and recycling bins were integrated into the design to promote proper waste management and encourage environmental stewardship throughout the forest. These elements are designed to complement the active and passive recreation zones and the ecological education areas, creating a harmonious balance among leisure, learning, and conservation.

#### 2.4.1. The Ecological Education Area

**Figure 16:**
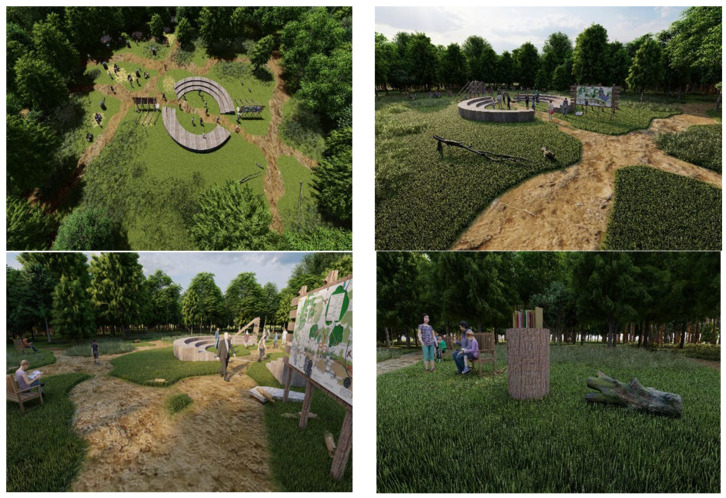
Ecological education area.

As the forest ecosystem, comprising a diverse range of flora and fauna, serves as an ecological catalyst for profound, awe-inspiring, and meaningful nature experiences, we designed an Ecological education area within the Green Forest as a valuable opportunity to connect the community with nature and to cultivate learning in the open air, as illustrated in Figure 16. We conceived this space as an outdoor University of Life Science, designed for learners of all ages, from preschool children to experienced researchers, who seek to study the forest ecosystem directly within the forest itself. The focal point of the composition is an open-air amphitheater, a proper space for educational presentations, performances, cultural activities, and workshops. Adjacent to the amphitheater, we proposed a reading area, equipped with benches and tables suitable for outdoor study and reflection. This space will include a thematic selection of books on the natural sciences with varying levels of complexity, addressing all age groups, as well as forest-inspired stories rooted in the local cultural landscape, thereby stimulating curiosity and engagement among visitors of all ages. The intention is that, through organized reading sessions, the space will promote critical thinking, creativity, and dialog within the community. To better understand Green Forest’s biodiversity, we have proposed two informative panels that highlight the unique characteristics of its flora and fauna. These panels will present information on the taxonomic classification, origin, distribution, and morphology of selected species, as well as their ecological importance within the urban landscape. They will include illustrations and graphic elements depicting trees, shrubs, herbs, insects, birds, and mammals that inhabit Green Forest, highlighting each species’ role in maintaining a healthy, balanced ecosystem. By developing this educational recreation area, we aim to create a functional space that inspires respect for nature, stimulates scientific curiosity, and fosters a desire to learn. In doing so, the area will become a community gathering place where every visitor can connect with the forest, gain new knowledge, and enjoy the present moment.

#### 2.4.2. The Passive/Transit Zone

**Figure 17:**
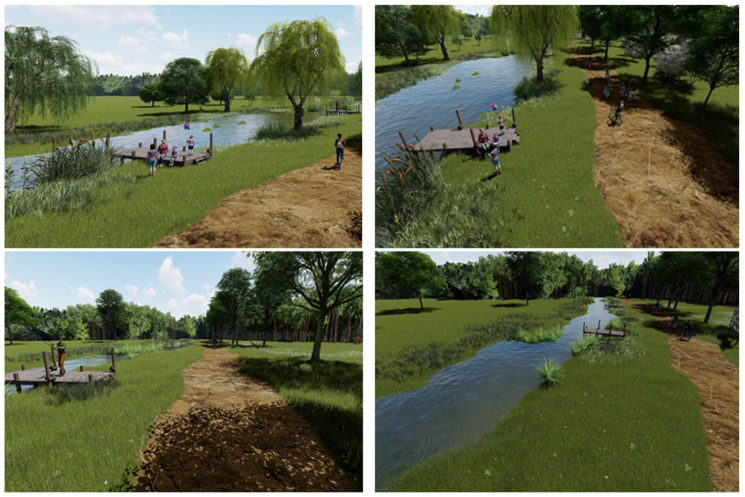
The Passive/Transit zone.

The Passive/Transit zone represents a forest route along a sector of the Green Forest—Behela River—Bega River green corridor, which forms a particularly valuable and unique green–blue area within Timișoara [37]. Located in the forest’s interior, it offers extensive vegetation, diverse natural landscapes, and clean, high-quality air. It was designed as a multifunctional space that accommodates long-distance activities such as walking, running, and cycling, as well as passive forms of recreation, including landscape appreciation, wildlife observation, birdwatching, picnicking, and interaction with the river environment. Consequently, we proposed seating areas in the form of wooden decks, enabling visitors to explore the Behela River’s aquatic ecosystem and benefit from its ecological and sensory qualities, for instance by meditating near the water or observing its acoustic and visual characteristics, as shown in Figure 17. Information boards and trail signage were also included in our proposal to inform visitors about local biodiversity and ecological processes. Certain signs indicating orientation and location were mounted on wooden logs, while others, featuring botanical illustrations of native plant species, invited visitors to “Find me,” encouraging an exploratory and interactive engagement with the Green Forest.

#### 2.4.3. The Active Recreation Zone

**Figure 18:**
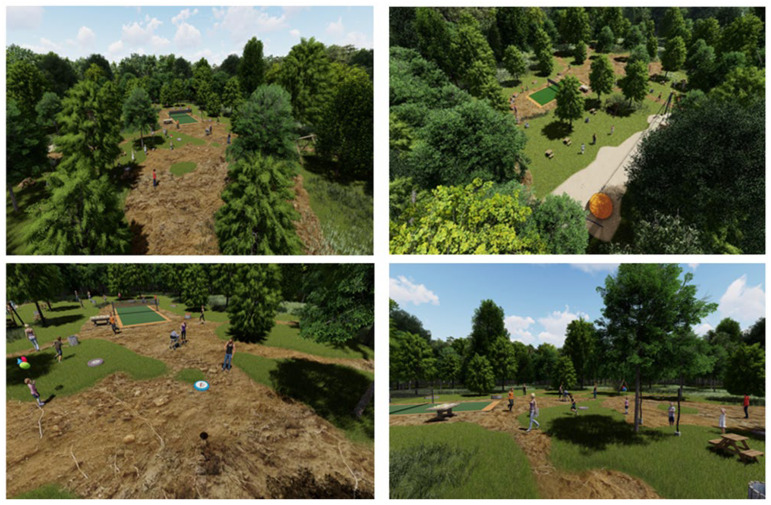
The Active recreation zone.

We conceived a family-friendly space within the Green Forest for various recreational activities, as shown in Figure 18. One of the main attractions is a zipline constructed from durable materials and designed in accordance with safety standards, offering a unique and dynamic experience with clearly marked, easily accessible takeoff and landing areas. In addition, we proposed several activity zones, including a badminton court, an area for frisbee play, and climbing structures, balance beams, swings, and seesaws to support a diversity of physical and social interactions. To complement the recreational experience, the design includes urban furniture such as benches, recycling bins, wooden decks, and picnic tables, as well as ecological toilets to ensure visitor comfort and promote responsible waste management. By creating a pleasant, accessible, and well-equipped environment, the project encourages a healthy and active lifestyle while supporting environmental protection and fostering respect for the forest.

## 3. Discussion

Green and blue infrastructures constitute a vital component of contemporary metropolitan ecological networks, contributing significantly to environmental quality, urban resilience, and the provision of essential ecosystem services. Green Forest holds a crucial role in advancing the Sustainable Development Goals within the Timișoara Metropolitan Area. As an integrated social–ecological system, its structure, functions, and resilience are shaped by the region’s social, economic, and institutional dynamics.

### 3.1. Tree Species Diversity in the Green Forest

Our findings confirm the high tree diversity of Green Forest, with 24 tree species representing 16 genera, including 18 autochthonous and 6 allochthonous species, a richness sustained by the widespread presence and structural dominance of native taxa such as *Quercus robur* and *Fraxinus excelsior*.

The analysis demonstrated that the mean age of *Quercus robur* (87 years) and the large average diameter (DBH 39.27 cm) observed in the majority of plots (Figure 1, Figure 2, Figure 3, Figure 4, Figure 5, Figure 6, Figure 7, Figure 8, Figure 9, Figure 10, Figure 11 and Figure 12) classify these individuals as essential components of the urban canopy. Such mature, large-diameter trees are disproportionately valuable in terms of carbon sequestration and cooling effect compared to younger, smaller individuals, aligning with findings by Fauziah et al. (2024) regarding the critical role of structurally diverse stands in enhancing carbon stocks [38].

Yao et al. (2023) demonstrated that evolutionary history and past ecological processes shape differences in species composition, thereby influencing the formation and maintenance of beta diversity within forest communities [39]. In this context, the Green Forest, comprising both coppice stands and composite coppice stands, reflects a historically shaped forestry structure. Over time, many of these stands have been artificially regenerated by introducing additional tree species, including both autochthonous and allochthonous taxa. Such interventions have contributed to the forest’s present heterogeneity, with implications for forest ecosystem structure at the species composition level, ecological functioning, and potential resilience.

However, a critical finding from the dendrometric analysis is the predominantly low stand consistency (C), which was recorded as ‘degraded’ in the majority of plots for key species such as *Quercus cerris* (93.88%), *Acer campestre* (100%), and *Fraxinus excelsior* (77.55%) (Section 2.2). This low density suggests a highly heterogeneous, open-canopy environment, which, while favoring light transmission and understory development, also indicates potential vulnerability to external pressures or management deficits. The structural predominance of low canopy density across 77–100% of plots is mainly the result of anthropogenic influences and historical urban land-use pressure, including forest fragmentation, edge effects, and recreational overuse, which are typical for long-established urban forests. This open-canopy condition is therefore interpreted as a legacy impact of urban disturbance, and not as evidence of appropriate silvicultural management or uniform natural succession processes. We further note that over the past 20 years, increased tree mortality and the presence of numerous dry and declining trees have also been influenced by the negative impacts of climate change, including prolonged summer droughts, heat stress, and irregular seasonal water availability, affecting soil moisture accessibility. These climatic pressures have contributed to local canopy thinning, but their expression remains gap- and microsite-dependent, rather than a plot-wide silvicultural design outcome.

The multivariate analysis further elucidated this composition variability. The Principal Component Analysis (PCA) isolated the contribution of rare and mature species (Ca. sat, Ti. tom, Pi. abi, Ps. men, P. syl, and T. bac) along the first dimension (explaining 25.6% of the variability), and segregated these unique structural features into six minor, isolated clusters (2–7). Conversely, the majority of the forest falls within Cluster 1, characterized by the common, low-density species defining the second dimension (U. min, R. pse, Q. rob, and A. cam). This pattern confirms that the forest structure is not uniform but is composed of a large, homogeneous matrix of low-density forest interspersed with highly localized micro-sites of ecological rarity and high structural complexity, likely remnants of older, more intensely managed stands or specific planting efforts. As an uneven-aged stand containing trees across all developmental stages, from seedlings to mature and senescent individuals, it represents a largely underrecognized yet substantial reservoir of biodiversity and ecological complexity, indicating ongoing regeneration processes and a relatively stable forest dynamic. Understanding the structure of the Green Forest stand is therefore essential for determining appropriate intensities of tending and management cuttings, as well as for identifying priority trees for removal to support stand health and adaptive capacity. The presence of genera such as Quercus, Fraxinus, Acer, and Ulmus across most plots suggests that species in these genera are well adapted to current site conditions. Vertically, their tree crowns form multiple layers. This structural heterogeneity enhances habitat diversity, supports species persistence, and enriches the city’s landscape architecture, positioning Green Forest as a key asset in shaping Timișoara’s long-term ecological sustainability.

As shown by Fauziah et al. (2024), forests with high species diversity within vegetation communities play a critical role in enhancing carbon stocks, underscoring the ecological value of structurally diverse stands such as those found in the Green Forest [38]. However, this ecological potential is threatened by climatic pressures. Chivulescu et al. (2025) indicate that extended heat waves exacerbate drought stress, elevate tree mortality rates, and drive shifts in community composition, highlighting the vulnerability of forest systems under intensifying climate change [18].

Particular attention should be directed toward *Quercus robur*, a species shown to be highly sensitive to the timing of summer drought and prone to growth decline under future warming scenarios [40,41,42]. The finding that the dominant and structurally most valuable species, Quercus robur, is highly represented in mature age classes (mean age: 87 years) must be directly addressed in Green Forest management planning by including species diversification through the introduction or expansion of drought-tolerant native species with contrasting water-use strategies (*Pinus nigra*, *Pinus sylvestris*, *Corylus avelana* or *Cornus mas*, shallow-rooted *Malus sylvestris* or deep-*rooted Salix alba*, *Salix fragilis*, and *Acer platanoides*), assisted migration [43] of a native drought-tolerant species, Quercus pubescens (‘assisted gene flow’ referring to the transfer of seed provenances to a new location—Green Forest—within the present distribution range). As this species is demonstrably prone to growth decline under future warming scenarios, the low stand consistency observed for most species (Section 2.2) becomes a compounded risk factor. Lower density can increase ground-level heat and drought exposure, particularly for sensitive mature individuals, but also can decrease the competition between young oaks in natural regeneration, making targeted silvicultural interventions crucial for maintaining the health of these vital carbon assets and for optimistic prognoses for the future of this species in the center of its current distribution range.

*Robinia pseudoacacia* is currently confined primarily to planted ornamental and landscape contexts, where its aesthetic attributes and prolific flowering contribute to the cultural and visual identity of the urban forest. Across all sampled plots, the species exhibits a mean age of 60.33 years (SD = 21.25), indicating a largely mature population with limited recent establishment. Only one plot (6.67%) contains individuals with a mean age of approximately 20 years, whereas the majority of plots support trees averaging between 30 and 60 years (53.33%) or 60 and 90 years (33.33%); a single plot (6.67%) includes individuals with a mean age of approximately 110 years. This age structure suggests that R. pseudoacacia persistence is currently driven by historical planting rather than active natural regeneration. Nevertheless, given the species’ well-documented invasive potential, any future recruitment into regeneration layers should trigger targeted monitoring and the implementation of adaptive, site-specific management measures to prevent uncontrolled spread.

A primary limitation of this study lies in the scope of the dendrometric analysis, which was restricted to tree populations and did not integrate shrub communities. Furthermore, neither herbaceous vegetation nor faunal communities were considered, which limits the ecological comprehensiveness of the findings. Consequently, the observed structural variability, particularly the low stand consistency, could not be fully correlated with understory development or overall microhabitat heterogeneity. Furthermore, while PCA and cluster analysis identified structural patterns, the proportion of explained variance by the first two dimensions (34.2%) suggests that other unmeasured environmental or historical factors contribute significantly to the total variability of the Green Forest stand. While the first two principal components explain 34.2% of the total variance, this reflects the high structural and interspecific complexity of the Green Forest ecosystem. In ecological studies involving diverse multi-species stands, such values are indicative of strong underlying patterns despite the inherent variability of biological data [26,44,45]. Future research incorporating edaphic factors and light transmission indices could further refine the understanding of the drivers behind this structural diversity. The multivariate analysis (PCA) revealed a clear distinction between the dominant native matrix, primarily composed of *Quercus robur* and *Fraxinus excelsior* (autochthonous species), and localized clusters of high ornamental and conservation value, such as *Castanea sativa* and *Pseudotsuga menziesii* (allochthonous species). Identifying these structural ‘islands’ is essential for landscape design, as they serve as biological landmarks for the proposed functional zones.

The present study focuses on providing a detailed descriptive baseline of the Green Forest’s dendrometric structure to inform urban planning and landscape design. While inferential statistical tests were not the primary focus of this architectural framework, they represent a crucial next step. Future research by the authors will build upon this baseline to explore species-specific ecological drivers, biodiversity indices (α, β, γ), and carbon sequestration potential using advanced remote sensing and inferential modeling.

To obtain and manage more accurate data for the Green Forest stand, we propose a novel approach utilizing airborne Light Detection and Ranging (LiDAR) multispectral and hyperspectral data for automated forest stand delineation, a method shown by Xiong et al. (2024) to enhance practical performance and forest management efficiency by outperforming manual delineation by 7.31% and multi-scale segmentation by 2.13% [46]. Moreover, following the recommendations of Meca I.A. et al. (2025), we propose integrating QGIS and QField into an optimized workflow that enhances, rather than bypasses, existing methodologies [47]. This integrated approach represents a pragmatic and incremental step toward digital twin models of urban green infrastructure, where ground-based measurements and spatially continuous remote-sensing data are jointly used to create a dynamic and living digital representation of Green Forest’s ecological assets. According to Şmuleac, A. et al. (2015), data processing with LiDAR and satellite images facilitates the creation of 3D models, the detection of the soil surface, data filtering, and vegetation classification [48]. This will maximize the incorporation of stand structure information, including tree height, canopy closure, and tree species details, and will lead to more efficient stand delineation in the Green Forest management inventory. Moreover, Dragomir L.O. et al. (2025) demonstrated that the hybrid methodology incorporating UAV photogrammetry, terrestrial SLAM LiDAR, and GNSS-based ICP validation produces highly accurate and efficiently generated cadastral plans [49]. In this context, the approach may be effectively applied to Green Forest mapping and monitoring, enabling precise boundary delineation, high-resolution spatial characterization, and reliable detection of temporal changes, thereby supporting sustainable forest management.

Our results address only tree species diversity rather than entire ecosystem biodiversity, while acknowledging the well-recognized contribution of the Green Forest to Timișoara’s urban biodiversity. Deepening our understanding of the mechanisms of species coexistence within Green Forest communities will provide critical scientific evidence to enhance ecosystem stability while balancing its ecological, aesthetic, and social functions. Future research should include a complete shrub and herb layer survey to refine the classification using formal syntaxonomic units (e.g., alliances and associations of Central-European and Pannonian mixed oak forests).

### 3.2. Conceptual Landscape Architecture Proposals for the Green Forest

In the context of unpredictable global warming impacts, the planning of Green Forest increasingly emphasizes forest health and ecosystem resilience, with the aim of safeguarding ecological integrity while simultaneously enhancing tourism attractiveness and generating sustainable economic, ecological, and social benefits, in line with good practices identified in comparable European urban forests [50,51]. To support this objective, Green Forest is conceptualized as a functional urban forest park capable of delivering provisioning, regulating, cultural, and supporting ecosystem services, with a landscape architecture framework explicitly aligned with residents’ needs, an aspect recognized as critically important in effective urban forest planning and management [52]. In response to pressing global challenges such as biodiversity loss and the climate crisis, we propose implementing Nature-Based Solutions (NBSs) that address ecological concerns while meeting human needs. By advancing three main approaches to designing sections of the Green Forest stand, namely the Ecological Education Area, the Passive/Transit Zone, and the Active Recreation Zone, we aim to enhance citizens’ quality of life by offering diverse opportunities for meaningful and enriching experiences within the forest ecosystem, while placing particular emphasis on nature conservation and recreational functions and actively promoting a strong local forest identity.

Located in a densely built urban area of Timișoara, Green Forest serves as a refuge for species with specific ecological requirements and is an important source of biodiversity for the surrounding green infrastructure within Timișoara’s urban matrix. Therefore, we propose creating a Transit zone along the Behela River, which flows through Green Forest and serves as a green corridor, strengthening the connection between the forest and the belt of green spaces along the Bega Canal. Enhancing connectivity between the forest and the urban landscape is essential to facilitate species distribution and support their successful establishment across the city’s green infrastructure. Our proposal is inspired by well-established European urban forest models, notably the Vienna Woods bordering the Austrian capital. This forest, dominated by European beech (*Fagus sylvatica* L.) and including a UNESCO Biosphere Reserve, delivers a wide range of ecosystem services to Vienna’s urban population, including recreation, human well-being, air purification, climate regulation, freshwater provision, soil erosion control, and timber production [53]. These services are actively sustained through targeted forest management strategies designed to balance ecological integrity with social and economic objectives.

As social perceptions and recreational needs related to Green Forest have recently been examined by Crețan et al. (2024), whose survey highlights strong demand for age-appropriate activities, low-impact recreation (e.g., walking, jogging, cycling, picnicking), and improved basic infrastructure (e.g., litter bins, seating, drinking water, and toilets), while emphasizing the importance of preserving forest tranquility, our proposals are deliberately aligned with these expressed community needs [54]. Consequently, the Active Recreation Zone is conceived as a family-friendly area within Green Forest, designed to accommodate a range of recreational activities, including a zipline, badminton court, frisbee play areas, climbing structures, balance beams, swings, and seesaws, thereby supporting diverse physical and social interactions. These facilities are intended to be constructed from durable materials and integrated sensitively into the forest setting, promoting healthy and active lifestyles while ensuring environmental protection and fostering respect for the forest ecosystem. An inspiring model is the historic forest Bois de Boulogne, whose interior is characterized by a diversity of landscapes that accommodate urban leisure, sports, and exhibition functions, including walking paths, picnic areas, a zoo, meadows, ponds, streams, islands, and designated sports fields [55]. Another example of good practice is represented by Oslo’s urban and periurban forests, which provide a wide range of valuable ecosystem services arising from the interaction of ecological, socio-cultural, and economic factors that reflect the forests’ history, cultural significance, and governance framework. These forests play a central role in everyday urban life, serving as recreational areas for approximately 86% of the city’s residents [56]. Drawing on good-practice examples from Oslomarka, we adapt these principles to the local context of Green Forest. In this respect, we propose the Ecological Education Area as an outdoor “University of Life Sciences” within Green Forest, designed to engage learners of all ages and to function as a platform for open-air, community-based learning that strengthens local identity and fosters a strong sense of place.

All existing Green Forest patches are considered valuable in their entirety, regardless of species composition, as they already function as integral components of Timișoara’s official green infrastructure. The diversity of plant genotypes, expressed through their unique morphological characteristics, such as form, volume, and color, creates unity through diversity, a fundamental principle in landscape architecture. Accordingly, our proposal follows the principle of harmony by introducing equipment and facilities constructed from materials that are visually compatible with the forest environment. By creating seating areas in close proximity to aquatic ecosystems, we aim to enhance experiential landscape design by enabling visitors to engage directly with natural soundscapes, such as flowing water, insects, and bird calls, thereby activating multiple sensory dimensions of perception, including sight, smell, hearing, and respiration, and fostering a deep sense of immersion and embodiment within the forest ecosystem. The network of long-circuit paths provides visitors with opportunities to explore Green Forest and to become active participants in its landscape.

In order to highlight and preserve the natural beauty of Green Forest—particularly its valuable species and habitats—the application of responsible forest stewardship is essential. This approach must address social dimensions and integrate modern forestry practices, including active conservation measures, the retention of habitat trees to support biodiversity, and dead wood management.

Safeguarding rare and threatened species such as *Neottia nidus-avis*, *Cephalanthera damasonium*, *Cephalanthera longifolia*, *Epipactis helleborine*, *Platanthera bifolia*, *Epipactis microphylla*, and *Ruscus aculeatus*, identified by Chiricheș T. (2013) within the Green Forest, is central to maintaining the ecological integrity and functional resilience of this ecosystem [57].

Adopting the measure of maintaining biodiversity trees alongside retaining dead wood would exert a highly favorable influence on overall biodiversity. Furthermore, such practices would enhance the aesthetic quality of the forest landscape.

Biodiversity trees create ecological continuity between regeneration cycles, enhance the structural heterogeneity of the forest, and increase the volume of dead wood within the ecosystem. Biodiversity trees should be organized into representative groups of high ecological value and maintained until the end of their biological lifespan. These areas should then be progressively replaced by similar patches during regeneration cuttings. To maximize ecological effectiveness, such groups should be well distributed throughout Green Forest. Suitable candidates include large trees (with DBH > 50 cm) exhibiting decay (e.g., *Quercus robur*, *Quercus cerris*, or *Fraxinus excelsior*, as well as trees representing all 24 tree species occurring in the area), featuring a variety of cavities and structures such as woodpecker cavities, mold cavities in trunks, dendrotelms and water-filled holes (diameter > 20 cm), insect galleries and tunnels, along with large trees (particularly *Quercus robur* with DBH > 50 cm) belonging to decay classes 4 and 5 (number of trees per hectare as specified in the forest management plan), in order to provide essential habitats and microhabitats for a wide range of fauna and flora species.

Dead wood itself functions as a key stability factor in forest ecosystems, exerting major significance for several fundamental ecological components, including the return of organic material into biogeochemical cycles, the support of decomposer communities, and the provision of habitat for insects, fungi, plants, birds, amphibians, and mammals. Dead wood retention in the study area has recently been shaped by an accelerated tree mortality phenomenon observed over the last two years, during a period when no wood extraction was carried out in Green Forest. Based on current evidence, this mortality is likely associated with prolonged drought conditions in the region, which caused the drying of the Behela stream and its tributaries, combined with the cumulative impact of numerous groundwater drillings conducted over the past 30 years in the surrounding residential zone, which now entirely encloses the forest. These pressures appear to have altered local hydrological conditions, contributing to stress-induced decline. Recent field observations (unpublished, personal monitoring data) indicate substantial dieback, particularly affecting very old, large trees of *Quercus robur* (estimated age > 100 years, height > 25 m, DBH > 50 cm), along with ongoing ash (*Fraxinus excelsior*) dieback. This process has resulted in high and spatially widespread volumes of dead wood, currently estimated to range between 20 m^3^/ha and 150 m^3^/ha across the forest area. These mortality-generated volumes already encompass advanced decay stages (classes 3–5) and occur in a well-distributed spatial mosaic, creating suitable conditions for: establishing quantitative baselines for future monitoring, applying adaptive silvicultural and habitat-based management, and implementing volume-per-hectare retention targets aligned with the forest management plan.

All these aspects may be presented and discussed within the Ecological Education Area that we propose to establish, thereby providing a coherent framework for their protection, management, and long-term integration into the forest’s conservation strategy.

Ultimately, integrating detailed dendrometric assessments with the proposed landscape architecture framework provides a robust, evidence-based strategy for the Green Forest. When implemented within a framework of responsible forestry practices, the Ecological Education Area, Passive/Transit Zone, and Active Recreation Zone can contribute to positioning the Green Forest as a managed ecosystem that enhances Timișoara’s resilience, biodiversity, and urban quality of life.

## 4. Materials and Methods

### 4.1. Site Description and Data Collection

**Figure 19:**
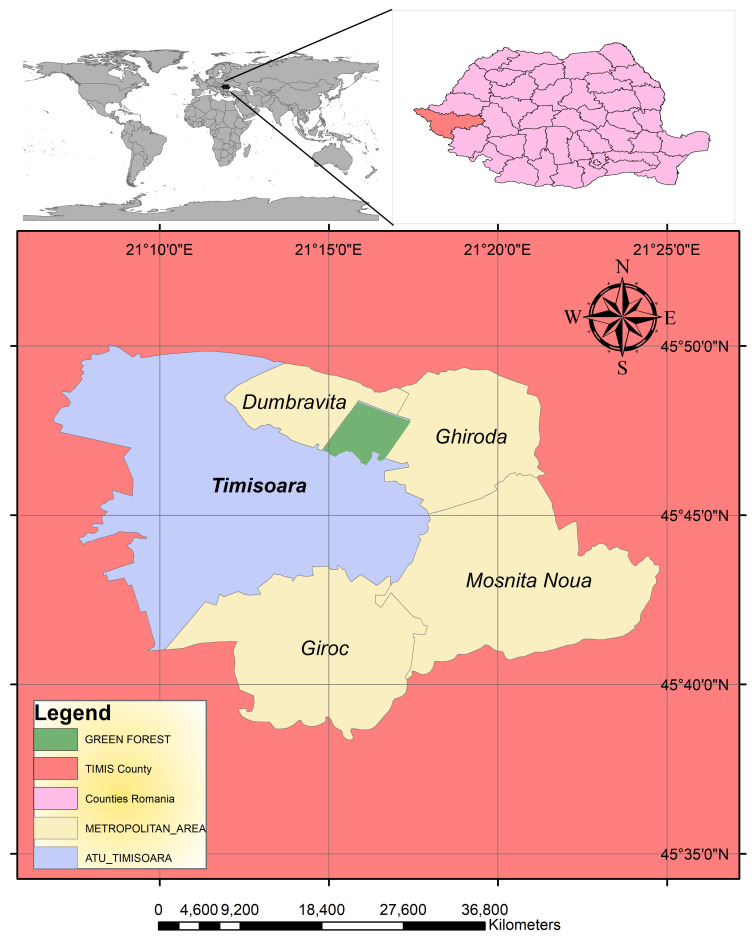
Timișoara metropolitan area.

Timișoara, located in the Western Romanian Plains within Timiș County (45.75372° N; 21.22571° E) (Figure 19), is an interconnected regional center currently experiencing continuous expansion and metropolitan transformation. Over the past two decades, the city’s urban boundaries have steadily extended, with neighboring localities—such as Dumbrăvița, Giroc, Moșnița Nouă, and Ghiroda—becoming integral components of its metropolitan ecosystem. This territorial growth has been fueled by economic development, foreign investment, infrastructure expansion, and population migration toward periurban areas. Urbanization threatens local biodiversity by degrading and fragmenting natural habitats and replacing them with gray infrastructure such as buildings and roads, which in turn contribute to higher levels of traffic-related air, soil, and water pollution [58,59]. Moreover, although the climate is warm and temperate—with an annual mean temperature of 13.2 °C and an annual average precipitation of 649 L/m^2^ unevenly distributed [60,61]—the results of a recent study by Micu et al. (2024) [62], quantifying the changing signals in the combined heat hazard (CHH) associated with concurrent hot days (HD—maximum temperature above 30 °C) and hot nights (HN—minimum temperature above 20 °C), reveal consistent geographical patterns in CHH change signals across both present and future climates in Timișoara. The city is projected to experience substantial increases in both the frequency and duration of CHH events, with values nearly doubling by 2050 and rising even further by 2100 [62].

In response, the Timișoara municipality is committed to preserving and expanding green and blue infrastructures, including Green Forest, within the urban landscape. This approach aligns with Croci et al. (2008), who suggest that woodlands are an effective means of promoting biodiversity conservation in towns [63].

**Figure 20:**
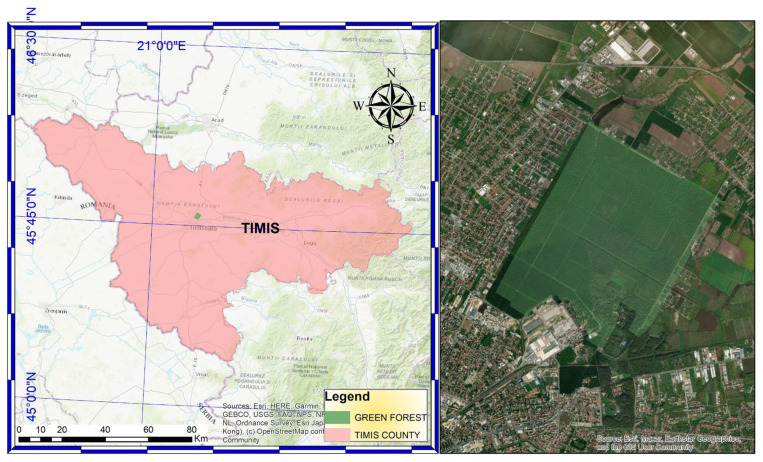
The Green Forest as part of Timișoara’s green infrastructure.

Green Forest (45.75372° N; 21.22571° E) (Figure 20) covers approximately 700 hectares in the northeastern part of Timișoara. Of this, 519.84 hectares were transferred to the Municipality of Timișoara by Government Decision No. 790/24.09.2020, reassigning the land from the public domain of the State and the administration of the National Forest Administration—ROMSILVA. Additionally, 60.74 hectares of land with other uses, including parcel boundaries, areas designated for game management, and non-productive land, were included in the transfer. Furthermore, over 100 hectares were returned to the Municipality of Timișoara through several successive actions in accordance with relevant legislation. As a result of these transfers, the green space per capita in Timișoara increased to approximately 38 sq m. Due to the Local Green Spaces Register currently being in progress, this information is subject to updates.

**Figure 21:**
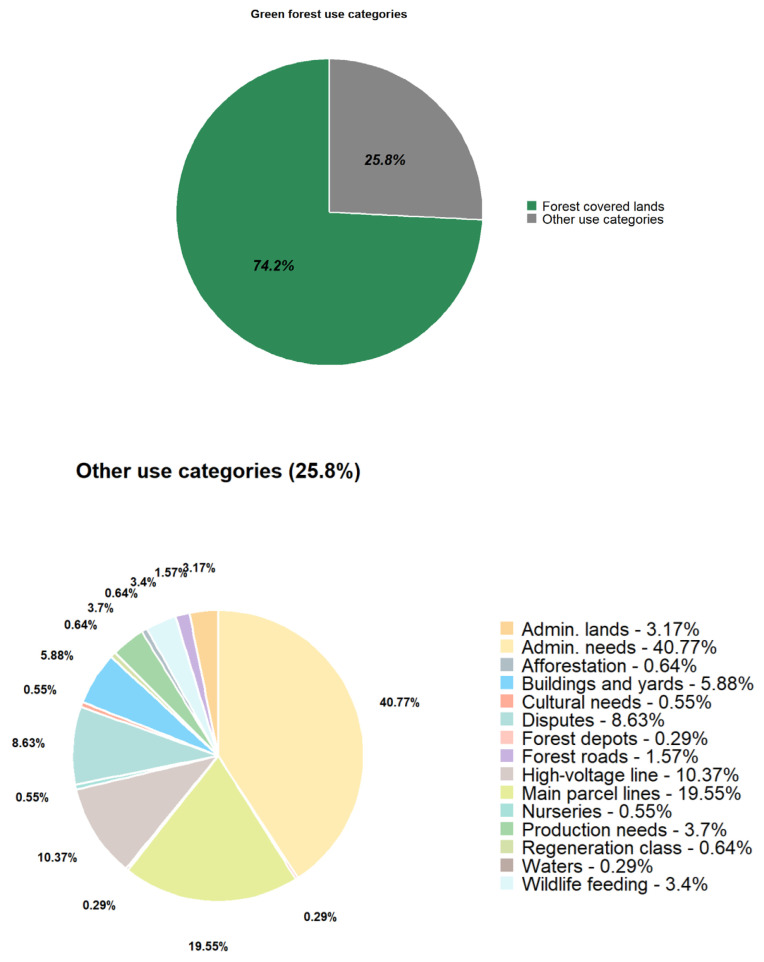
Green Forest fund area by use category.

The Green Forest land-use categories and management units, as defined in the 2018 management plan for Production Unit VIII (Forest District Timișoara, Forestry Directorate Timiș), are shown in Figure 21.

The Green Forest is a semi-natural lowland deciduous woodland shaped by more than 160 years of documented human-mediated regeneration and silvicultural management. Historical records indicate continuous structured forest stewardship beginning with the first official management plan in 1860, followed by revisions in 1894, 1908, 1926, and 1947. Early management objectives prioritized game-oriented land use, recreational hunting, and landscape accessibility, factors that indirectly influenced forest structure, species selection, and spatial organization of tree assemblages. After the nationalization of forest lands in 1948, the area became part of the national state patrimony and entered a long-term state-coordinated management framework administered by the Timișoara Forestry District. Subsequent forest management plans (1955, 1968, 1978, 1988, 1998, and 2008) guided silvicultural operations, stand regeneration methods, species introductions, and the spatial structuring of vegetation communities that define the present-day forest.

Past silvicultural treatments included coppicing and composite coppice fellings, practices that strongly contributed to the predominance of even-aged stands across the landscape. In areas managed through composite coppice systems, remnant overstory trees, primarily pedunculate or sessile oaks (*Quercus* spp.) and European ash (*Fraxinus excelsior*), persist from pre-treatment generations, providing ecological continuity and localized structural complexity.

Several non-native tree species were introduced, notably red oak (*Quercus rubra*) and black walnut (*Juglans nigra*), reflecting a lasting anthropogenic influence on forest composition and successional pathways. The younger forest layers are dominated by field maple (*Acer campestre*) and European ash (*Fraxinus excelsior*), species typical of lowland mixed deciduous plant communities in Central and Eastern Europe. These species contribute substantially to the structural and functional diversity of the regenerating understory and sub-canopy, forming assemblages characteristic of mesic, eutrophic plains forests. Many stands originated from artificial regeneration techniques, including direct seeding and planting, resulting in a vegetation mosaic influenced by both ecological site conditions and historical human design.

This legacy is also reflected in the forest’s ride network, linear clearings that increase light availability along edges, unintentionally supporting microhabitats for early-successional and ecotonal plant species. Although hunting was the forest’s original primary function, historical sources confirm that recreation and landscape aesthetics were also considered, creating a multifunctional land-use context that influenced vegetation layout. Archival sources identify the present site of the Forestry High School as a former hunting castle, supporting the interpretation that long-term human presence influenced both forest access networks and patterns of tree establishment, as well as vertical woodland stratification.

Overall, the Green Forest represents a semi-natural ecosystem where contemporary plant community structure, age distribution, and species composition are strongly linked to a long history of managed regeneration, selective species introductions, and anthropogenic spatial organization.

According to the most recent management plan (2018) for Production Unit VIII of the Green Forest, the surveyed forest patches can be assigned to three main forest community types: *low-productivity oak stands with Agrostis alba*, *medium-productivity mixed lowland oak forests of the plain*, and *typical mixed lowland oak forests of the plain*.

At the same time, the tree and shrub diversity of the Green Forest ecosystem is supported by a rich herbaceous layer, including species such as *Arum maculatum*, *Pulmonaria officinalis*, *Anemone ranunculoides*, *Corydalis cava*, *Campanula* sp., *Dactylis glomerata*, *Erythronium dens-canis*, *Ficaria verna*, *Fritillaria meleagris*, *Galium album*, *Geranium robertianum*, *Geum urbanum*, *Oryzopsis virescens*, *Primula vulgaris*, and *Prunella vulgaris*, which contribute to seasonal understory dynamics, overall plant biodiversity, and the aesthetic character of the forest, while noting that no Natura 2000 habitats have been formally identified within the Green Forest area.

Even though the Green Forest is strongly influenced by intense human activity, it maintains a well-represented biodiversity, including plants, birds, amphibians, mammals, and entomofauna, a finding consistent with the conclusions of Abrego and Medianero (2025) in their study on the diversity and composition of insect communities in four urban forest fragments near Panama City [64].

Despite being surrounded by buildings and roads, existing studies on the insect diversity of the Green Forest document a remarkable richness, including 635 species of butterflies [65], 35 species of dragonflies, two of which are of community interest [66], eight species from the family Cerambycidae [67], and a grading of the species *Stereonychus fraxini* was mentioned in 1958–1959 [68]. These findings suggest that the Green Forest ecosystem supports a wide variety of insect species, forming complex food webs that function as decomposers, pollinators, and natural enemies of other species, thereby maintaining high energy fluxes and providing important ecosystem functions.

Moreover, the Behela River flows through the Green Forest for 2.6 km. It constitutes a valuable green-blue corridor that connects the forest with the Bega Canal, along which it provides habitats for local fauna, constitutes a natural ecosystem with unique biodiversity, and contributes to maintaining ecological balance within Timișoara’s urban landscape [37,69].

### 4.2. Geographical Information System (G.I.S.) and Global Navigation Satellite System (GNSS) Technologies

The maps were created using Geographic Information Systems (GIS) technology, which is the most effective method for representing geospatial data [70]. The software used was ArcGIS version 8. Spatial data were represented in the WGS 84 projection system (Figure 1) and in the national Stereo 70 projection system (Figure 2).

### 4.3. Methodology for Data Collection for Forest Management Planning

For the statistical analysis, data were sourced from the most recent management plan (2018) for Production Unit VIII Green Forest, administered by the Forest District Timișoara under the Forestry Directorate Timiș.

According to Technical Norms no. 6/2022 for Forest Management Planning, the entire surface of the forest management unit (sub-parcel) is inspected. At characteristic points (at least 1 per 4 hectares), a sampling plot is installed, and 8–10 trees are measured and described. The species of each tree is noted, and the diameters and the height of the trees with the diameter close to the average diameter were measured. Measurement of two diameters at breast height (1.3 m) (DBH1, DBH2) at 1800 intervals, using a Haglöf Sweden AB aluminum tree caliper with a precision of 1 mm, was conducted. The average diameter at breast height (DBHm) was calculated. The heights of the trees with diameters close to the average were measured with Vertex equipment. The measured trees are marked with paint (the ones with measured heights are marked differently), and the sample plot location is recorded using a GPS. The three crown cover of the stand, its density, the proportion of participation of different tree species, and other stand characteristics are estimated at the stand level.

### 4.4. Statistical Analysis

All statistical analyses (including graphics) were performed using R Statistical Software (v4.4.1) [71]. Basic descriptive statistics for age, consistency, height, and diameter were used to characterize tree species diversity within the studied plots of the Green Forest in the Timișoara urban woodland. To explore the underlying structure within the consistency of tree species of the Green Forest of Timișoara urban woodland, Principal Component Analysis (PCA) was conducted. This technique reduces data dimensionality by identifying a smaller set of uncorrelated principal components that capture the majority of the variance in the original data. Subsequently, cluster analysis was performed on the derived principal components to group plots of the Green Forest of Timișoara urban woodland with similar characteristics.

### 4.5. Landscape Architecture Design Proposals-Methodology

Although the primary functions of the Green Forest are ecological (a diverse ecosystem), cultural (including the Forestry School Group, the Village Museum, and the anti-communist Resistance Monument in Banat), and recreational (cycling and walking paths), it currently lacks the requisite infrastructure and ecological management practices to qualify for urban forest park designation [72]. In this context, high-quality 3D visualizations were developed in ArchiCAD and Lumion to illustrate proposed designs for an ecological education zone, a passive recreation area, and an active recreation area within the Green Forest. The interventions are conceptual in nature and are meant to support decision-making and inspire future applied projects, rather than to serve as ready-to-implement policy instruments.

## 5. Conclusions

This study provided a comprehensive assessment of the tree diversity and structural complexity of the Green Forest, framing its ecological value within the context of Timișoara’s urban landscape architecture and strategic planning. The findings confirm the critical role of this forest park as a significant component of the city’s green infrastructure, enriching both biodiversity and quality of life.

Our analysis identified 24 tree species across 16 genera, demonstrating the high biodiversity of Green Forest, sustained by the structural dominance of mature native species, particularly *Quercus robur* (pedunculate oak) and *Fraxinus excelsior* (common ash). The mean age of *Quercus robur* (87 years) and the high average DBH (39.27 cm) underline the forest’s substantial capacity for long-term carbon sequestration and urban climate mitigation.

The multivariate analysis (PCA and Cluster Analysis) was instrumental in uncovering the stand’s underlying structural heterogeneity. While the forest is characterized by a prevailing matrix of low-density plots (often exhibiting “degraded consistency”), the analysis successfully isolated specific, highly localized micro-sites that host rare species and unique structural complexity. This evidence underscores the need to implement differentiated, adaptive forest management strategies that prioritize protecting these rare clusters and retaining valuable old-growth individuals.

Crucially, the research translates these ecological findings into practical application by proposing a functional three-zone landscape architecture framework: the Ecological Education Area, the Passive/Transit Zone, and the Active Recreation Zone.

The core contributions of this research are twofold:

Scientific Contribution: Providing the first detailed dendrometric and multivariate analysis of the Green Forest trees’ structure, establishing a foundational ecological baseline for future urban ecology research in the Timișoara Metropolitan Area.

Practical Contribution: Delivering realistic, evidence-based landscape design proposals (using ArchiCAD and Lumion) that maximize the forest’s ecological and recreational functions, directly supporting the objectives of the 2023 Action Plan for Timișoara, a Green City.

We conclude that, if implemented, the proposed interventions will significantly enhance Green Forest’s ecosystem services, foster deeper community engagement with nature, and position this blue–green infrastructure as a model for sustainable urban forest integration in Central and Eastern Europe.

## Figures and Tables

**Table 1:** Tree species recorded in the study, standard abbreviations, and origin status relative to Romania (autochthonous or allochthonous).

No.	Latin Name	English Name	Standard Abbreviation	Origin Status in Romania
1	*Quercus robur*	English oak	Q. rob	Autochthonous
2	*Quercus rubra*	Northern red oak	Q. rub	Allochthonous
3	*Quercus cerris*	Turkey oak	Q. cer	Autochthonous
4	*Quercus frainetto*	Hungarian oak	Q. frai	Autochthonous
5	*Fraxinus excelsior*	European ash	F. exc	Autochthonous
6	*Acer campestre*	Field maple	A. cam	Autochthonous
7	*Acer platanoides*	Norway maple	A. pla	Autochthonous
8	*Acer pseudoplatanus*	Sycamore maple	A. pse	Autochthonous
9	*Ulmus minor*	Field elm	U. min	Autochthonous
10	*Ulmus glabra*	Wych elm	U. gla	Autochthonous
11	*Robinia pseudoacacia*	Black locust	R. pse	Allochthonous
12	*Juglans cinerea*	Butternut walnut	J. cin	Allochthonous
13	*Juglans nigra*	Black walnut	J. nig	Allochthonous
14	*Prunus avium*	Wild cherry	P. avi	Autochthonous
15	*Carpinus betulus*	European hornbeam	C. bet	Autochthonous
16	*Gleditsia triacanthos*	Honey locust	G. tri	Allochthonous
17	*Pyrus pyraster*	Wild pear	Py. pyr	Autochthonous
18	*Tilia tomentosa*	Silver linden	Ti. tom	Autochthonous
19	*Castanea sativa*	Sweet chestnut	Ca. sat	Autochthonous
20	*Picea abies*	Norway spruce	Pi. abi	Autochthonous
21	*Taxus baccata*	European yew	T. bac	Autochthonous
22	*Pinus nigra*	Black pine	P. nig	Autochthonous
23	*Pinus sylvestris*	Scots pine	P. syl	Autochthonous
24	*Pseudotsuga menziesii*	Douglas fir	Ps. men	Allochthonous

Autochthonous—native to Romania; Allochthonous—introduced in Romania.

## Data Availability

The original contributions presented in the study are included in the article; further inquiries can be directed to the corresponding author.
